# Nuclear Resonance Vibrational Spectroscopy: A Modern Tool to Pinpoint Site-Specific Cooperative Processes

**DOI:** 10.3390/cryst11080909

**Published:** 2021-08-02

**Authors:** Hongxin Wang, Artur Braun, Stephen P. Cramer, Leland B. Gee, Yoshitaka Yoda

**Affiliations:** 1 SETI Institute, Mountain View, CA 94043, USA;; 2 Laboratory for High Performance Ceramics, Empa. Swiss Federal Laboratories for Materials Science and Technology, Überlandstrasse 129, CH-8600 Dübendorf, Switzerland; 3 LCLS, SLAC National Accelerator Laboratory, Menlo Park, CA 94025, USA;; 4 Precision Spectroscopy Division, SPring-8/JASRI, Sayo 679-5198, Japan;

**Keywords:** nuclear resonant vibrational spectroscopy, NRVS, Mössbauer spectroscopy, isotope-specific, site-specific, vibrational modes, phonons, partial vibrational density of state, PVDOS, inelastic scattering, high-resolution monochromator, HRM, real sample temperature, an almost zero background

## Abstract

Nuclear resonant vibrational spectroscopy (NRVS) is a synchrotron radiation (SR)-based nuclear inelastic scattering spectroscopy that measures the phonons (i.e., vibrational modes) associated with the nuclear transition. It has distinct advantages over traditional vibration spectroscopy and has wide applications in physics, chemistry, bioinorganic chemistry, materials sciences, and geology, as well as many other research areas. In this article, we present a scientific and figurative description of this yet modern tool for the potential users in various research fields in the future. In addition to short discussions on its development history, principles, and other theoretical issues, the focus of this article is on the experimental aspects, such as the instruments, the practical measurement issues, the data process, and a few examples of its applications. The article concludes with introduction to non-^57^Fe NRVS and an outlook on the impact from the future upgrade of SR rings.

## 1. Introduction

Nuclear resonant vibrational spectroscopy (NRVS) is a synchrotron radiation (SR)-based nuclear inelastic scattering (NIS) spectroscopy that measures the phonons (i.e., vibrational modes) associated with the nuclear transition [1–9]. NRVS has distinguished advantages and has been used by physicists, chemists, materials scientists, etc. for more than 25 years. While chemists call it NRVS, per historical reasons, physicists often call it by another name—nuclear resonant inelastic X-ray scattering (NRIXS) [10–13]. In this article, we present a scientific but figurative description of the experimental aspects of this wonderful modern tool to the potential future users working in various research fields.

### 1.1. NRVS Transitions and Selection Rules

**Figure 1.**
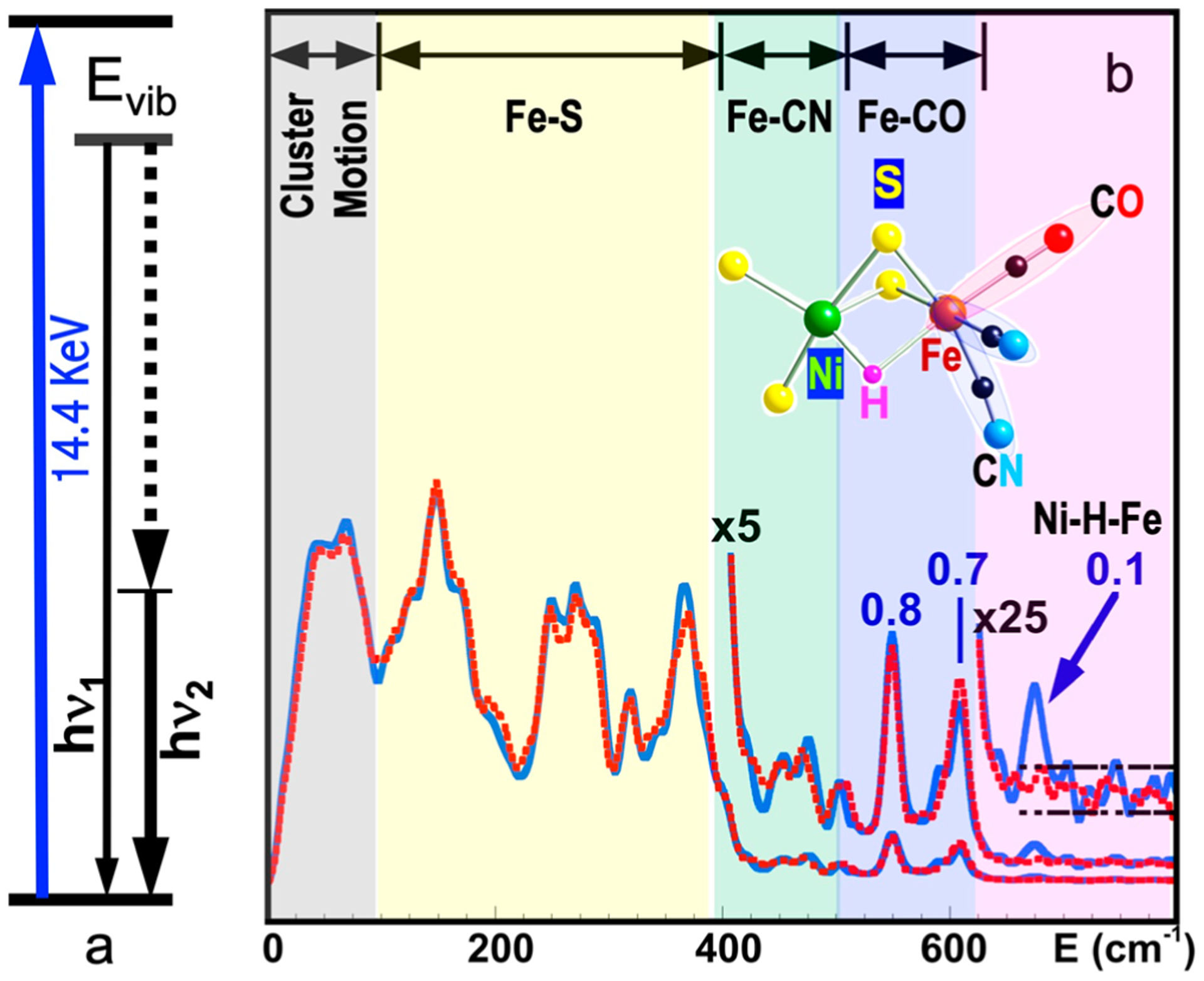
(**a**) An illustration of NRVS transitions; (**b**) ^57^Fe NRVS spectra for *DvMF* NiFe hydrogenase (reaction center structure = insert) in a hydride (blue) and deuterated sample (red). The peak assignments (bold black text) and peak counts per second (the blue numbers) are given for the hydride sample (blue curves).

Figure 1a shows the basic principle of NRVS transitions; while an incident X-ray beam scans through an interested energy region to cover the nuclear transition and the associated vibrations (e.g., E_1_ ~ 14.41425 keV for ^57^Fe), the nuclear back radiation can be monitored as the scattering energy (E_2_ = hν_1_). The total intensities collected from both the direct nuclear fluorescence at hν_1_ and the internally converted electron K shell fluorescence at hν_2_ vs. the vibrational energy E_vib_ = (E_1_–E_2_) is a raw NRVS spectrum. In this sense, it is similar to resonant optical Raman spectroscopy where vibrational information is extracted in an inelastic scattering from laser light excitation. An NRVS spectrum includes Stokes (creation of phonons) and anti-Stokes (annihilation of phonons) branches, although Figure 1a only illustrates the Stokes transitions. It is also similar to Mössbauer spectroscopy MS), except that MS measures the recoilless hyperfine interactions, while NRVS measures the recoiled transitions due to vibrations. The raw NRVS spectra can be transformed to partial vibrational density of state (PVDOS), which is independent of any experimental conditions and is calculable via theory-based mathematical simulations. Figure 1b provides an example of such PVDOS.

One of the central relations for any spectroscopy is the algebraic selection rule(s), which tell where the allowed transitions occur and, thus, what the spectral profile is like. For NRVS, there are major factors that govern its intensity distribution [14]. Since this article focuses on the experimental science, we omit the details of the formula deduction but summarize the conclusions instead.

As for any spectroscopy, the intensity for NRVS is proportional to the sample’s concentration and the population distributed on the ground state (e.g., the ground vibrational level). The ground-state population distribution is also a function of the ambient temperature T of the statistical ensemble.

Similar to other inelastic scattering process, NRVS also has a general ∝ 1/E_vib_ dependence that reduces its signal for higher-energy transitions.

Similar to Mössbauer transition, the NRVS signal level is proportional to the Lamb–Mössbauer factor (f_LM_ or LM factor for short). The LM factor (f_LM_) signifies the probability, i.e., whether or how likely a nuclear resonance will take place. For example, free atoms or gaseous samples have almost zero LM factors and have no nuclear resonance, neither Mössbauer nor NRVS. Isotope type and sample temperature T are also the factors that affect the LM factor. ^57^Fe is one of the few isotopes that show a significant nuclear effect even at room temperature (RT), while the nuclear transition for most other isotopes can only be observed at a cryogenic temperature. This poses a fundamental problem for *operando* investigations of materials, which function only at ambient or high temperatures. In addition, the LM factors depend on the molecular environment of the probed nuclei. For example, the iron in lithium iron phosphate undergoes changes in the oxidation state during de-lithiation, and so does its LM factor, which Aldon et al. have demonstrated recently [15].

If one assumes a harmonic vibrator inside a lattice or a molecule, then the whole transition *S*(*E*) can be separated into the nuclear resonant transition *δ*_Γ_(*E*), the single-phonon transition *S*_1_(*E*), and multiple-phonon transitions *S*_*n*_(*E*) as in Equation (1).


(1) $$S(E)=f_{LM}\left(\underset{\;}{\underset{\text{Mössbauer}}{\underset{︸}{\delta_{\text{Γ}}(E)}}}+\underset{\text{NRVS}}{\underset{︸}{S_{1}(E)+\sum_{n=2}^{\infty }S_{n}(E)}}\right)$$

For large *f*_*LM*_ (such as *f*_*LM*_(^57^Fe) = 0.8 at low temperature), the *S*_1_(*E*) will dominate the NRVS transitions: *NRVS* ~ *S*_1_(*E*).

The single-phonon transition or fundamental vibration *S*_1_(*E*) which we care the most for is proportional to the ‘mode composition factor’, $e_{j\alpha }^{2}$, as expressed in Equation (2):

(2) $$S_{1}(E)\propto e_{j\alpha }^{2}=\frac{m_{j}r_{j\alpha }^{2}}{\sum_{k}m_{k}r_{k\alpha }^{2}}$$

where $r_{j\alpha }^{2}$ is the mean square displacement and describes the displacement for a particular atom *j* in a particular vibrational normal mode α. Thus, the *NRVS* for a particular vibrational mode α is proportional to the mean square displacement of the isotope *j* in the nuclear transition (e.g., *j* = ^57^Fe), as illustrated in Equation (2). This defines the selection rule for NRVS, similar to the transition dipole moment in IR spectroscopy or the polarizability tensor in Raman spectroscopy. The formula also sets the foundation for a calculable PVDOS [2,14,16,17].

Although most NRVS experiments are conducted on powder samples or frozen solutions with random molecular orientations, there is additional anisotropy information, which can be gained when single crystal samples are examined. This may sound interesting for the condensed matter physics community, but every discipline interested in vibration properties should be interested in anisotropy of their systems. For example, NRVS will be different for an incident beam along three perpendicular directions in the sample (*x*, *y*, and *z*); for this case, there will then be three distinct mode composition factors, $e_{j\alpha ,x}^{2}$, $e_{j\alpha ,y}^{2}$, and $e_{j\alpha ,z}^{2}$, corresponding to the projection of the nuclear motion along *x*-, *y*-, and *z*-axes.

### 1.2. Historic Evolution of NRVS

As a nuclear scattering spectroscopy method, NRVS is related to traditional Mössbauer spectroscopy. In 1958, prior to his doctoral exam, Rudolf Mössbauer was the first to discover the recoilless nuclear resonant absorption (the Mössbauer effect) on ^191^Ir [18,19], followed by that on ^187^Re, ^177^Hf, and ^166^Er. Since then, the effect has been found in about 40 elements, 90 isotopes, and 110 nuclear transitions, with ^57^Fe at 14.4125 keV being one of the most useful and most researched transitions. Probing vibrations and dynamics through coupled nuclear transitions was also proposed almost immediately after the discovery of Mössbauer effect and explored in the early 1960s [20,21]. For laboratory-based Mössbauer spectroscopy, incident energies are tuned via Doppler effect by moving a radioactive source back and forth. Without difficulty, it is possible to provide an energy span for Mössbauer spectroscopy, which has a less than 100 neV in its energy span. However, in order to measure vibrations, an energy span of about 100 meV (or even 200 meV) is needed, corresponding to a speed to move the radioactive source at about 2 km/s (for ^57^Fe), six orders of magnitude faster than a typical Mössbauer experiment. Therefore, such a setup (for NRVS) is considered mechanically difficult and dangerous, as well as impractical. In short, an entirely different type of X-ray source is required for a NRVS experiment.

Synchrotron radiation (SR) is the electromagnetic radiation emitted by electrons when they travel along a curved trajectory at relativistic speeds (close to the speed of light where relativistic corrections determine the physics). SR at highly advanced facilities has many advanced properties including a high beam flux, a wide/adjustable energy range, a small beam size and a good beam collimation [22,23]. Using SR as a light source for nuclear resonance spectroscopies was proposed in 1974 [24] and first explored in the mid-1980s with a successful nuclear Bragg diffraction (a nuclear elastic scattering) in yttrium iron garnet material [25]. For comparison, a regular Bragg diffraction (very important in X-ray crystallography) is elastic electronic scattering. For this pioneering experiment, a nuclear Bragg monochromator was developed which contained two 15 μm thick single crystal films from an yttrium iron garnet (we believe this was Y_3_Fe_2_[FeO_4_]_3_) epitaxial grown on a gadolinium gallium garnet (we believe this was Gd_3_Ga_5_O_12_) substrate with 30 mm diameter. The iron garnet films were enriched to 88% with ^57^Fe [25]. In the mid-1990s, with most of the proposed fundamental issues explored and a few third-generation high-energy SR rings available, scientists at three SR centers near-simultaneously made successful observations of the recoiled nuclear inelastic scattering caused by vibrational dynamics [1,2,4]. These SR centers are the three current super SR centers on the globe, SPring-8, APS, and ESRF, which remain the major global hubs for nuclear resonance spectroscopies up to today.

After the initial observations, on the basis of the high-brightness third-generation SR and the continuous improvement of tunable monochromators with sub-meV resolution, use of NRVS has boomed. ^57^Fe NRVS, as well as various nuclear resonant scattering (NRS) spectroscopies, has attracted particular attention among physicists and geophysicists first because iron is an archetypal transition element and is a dominant component in the cores of the Earth and other terrestrial planets. Since 2001, ^57^Fe NRVS has enjoyed particular emphasis in inorganic biochemistry research, on various metalloenzymes such as myoglobin and heme systems [26–28], [NiFe] hydrogenase [7,9,29], [FeFe] hydrogenase [30,31], other hydrogenases [32], Mo-nitrogenases [6,33], nonheme systems [34], and various iron–sulfur systems [9,35–37]. Meanwhile, ^57^Fe NRVS application to other research fields, as well as NRS/NRVS for isotopes other than ^57^Fe, has also been evaluated and published [38–40].

### 1.3. NRVS Advantages

Advantages of a synchrotron-based Mössbauer method are the small beam size and very good beam collimation. This allows for many applications in chemistry, biology, materials science, geosciences, high-pressure physics, and many more, particularly because conventional nuclear methods require large amounts of sample, the provision of which can be a costly endeavor or even a technically impossible undertaking. Polarization of synchrotron radiation also allows for studying the magnetic structure of the specimen [41].

The most prominent advantages of NRVS include but are not limited to being able to extract site-specific information from highly complex systems (e.g., from biological molecules [5–9,42]), having an almost zero background [7,8], and having a theoretically calculable PVDOS [2,5,17,28].

**Figure 2.**
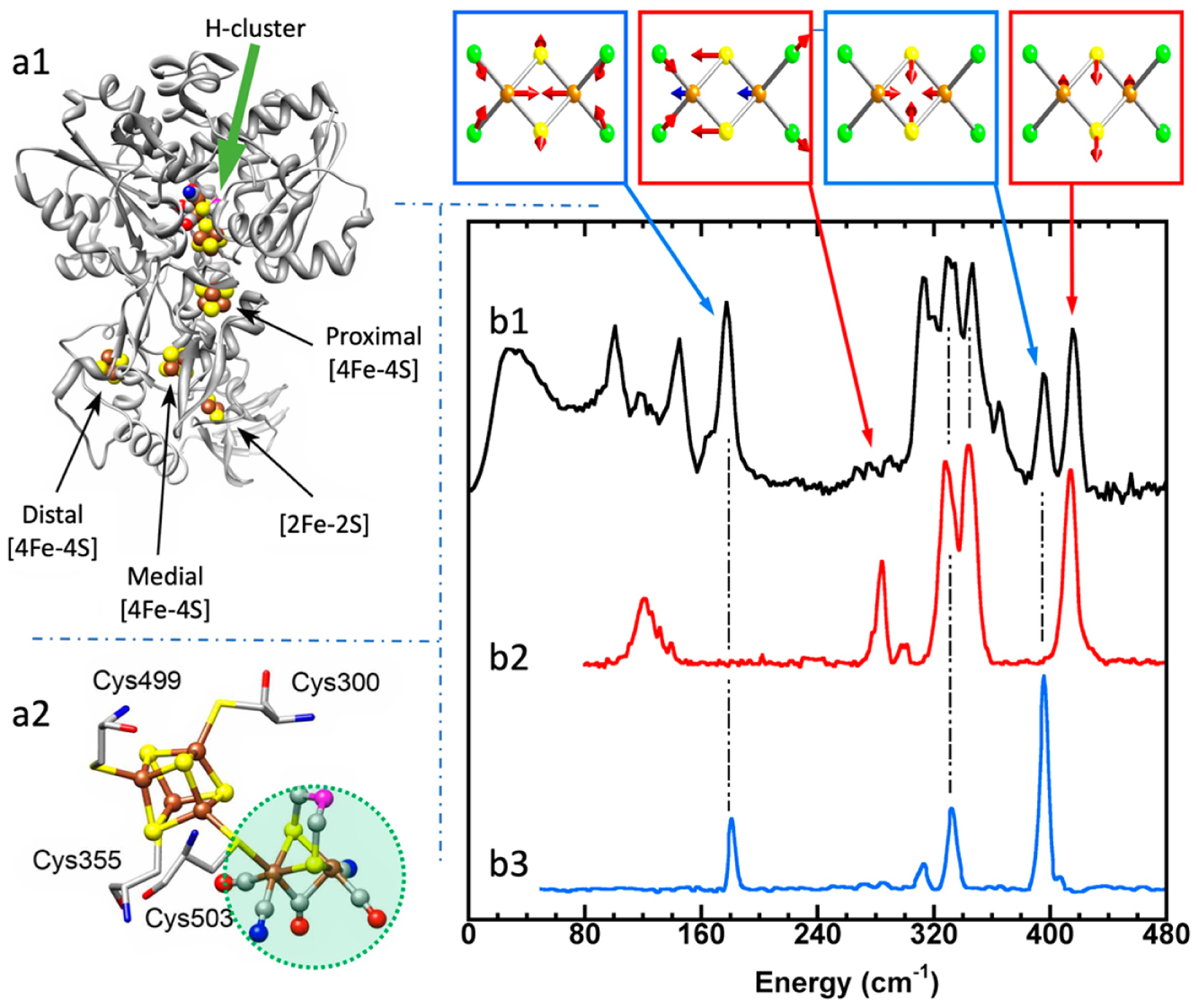
Left panel: structure for an [FeFe] hydrogenase (**a1**) and its reaction center (H-cluster, six irons) (**a2**). The green shaded area in (a2) is the reaction center [2Fe]_H_ subcluster (two irons). Right panel: NRVS-derived PVDOS (**b1**, black) along with IR (**b2**, red) and Raman spectra (**b3**, blue) for [Fe_2_S_2_Cl_4_]^=^. The top inserts above (b1) illustrate a few distinct vibrational modes for this complex.

Using ^57^Fe NRVS as an example, the NRVS selection rule (Equation (2)) excludes all the non-^57^Fe vibrations. For example, an [FeFe] hydrogenase enzyme such as the one in Figure 2a1 often has various iron–sulfur clusters in addition to its H-cluster (a2) which hosts the reaction center [2Fe]_H_ (a2, green circled and shaded) [30,43]. Nevertheless, we are now able to identify and label the two irons just inside the [2Fe]_H_ center or six irons inside the H-cluster with ^57^Fe while leaving other irons unlabeled [30,31,44,45]. This lets the corresponding NRVS spectra specifically represent the irons inside the [2Fe]_H_ center (or H-cluster). NRVS is, therefore, a perfect tool to pinpoint the site-specific information inside a complicated molecule, such as the various enzymes including hydrogenases and nitrogenases [46,47].

On the other hand, NRVS also has the ability to show almost all the ^57^Fe-related vibrations provided these vibrational modes have ^57^Fe in motion. Figure 2b is the NRVS-derived PVDOS (b1) along with IR and Raman spectra (b2, b3) for a complex ion [Fe_2_S_2_Cl_4_]^=^ [48]. In its total 18 vibrational modes, 16 of them have NRVS signals, leading to a rich PVDOS as shown in (b1). The top inserts illustrate a few distinct vibrational modes for this complex; all of them have NRVS signals while three of them have prominent NRVS peaks. In comparison, IR (b2, red) or Raman (b3, blue) spectra for this complex each only has a few transitions (peaks).

Due to the simplicity of the NRVS intensity distribution (Equation (2)), the pure molecule-based experiment-independent PVDOS can be obtained from the raw NRVS spectra, and their energy positions as well as intensities can be well reproduced via various theoretical calculations. Calculation of the NRVS is included in the ORCA quantum chemistry program package [49]. In contrast, IR or Raman calculations often involve assumptions about molecular properties such as trail dipole moments or polarizabilities in order to obtain an approximate molecular PVDOS. In the rest of this article, unless mentioned otherwise, NRVS spectra refers to PVDOS.

Using ^57^Fe as an example, the nuclear scattering energy can be precisely selected by the extremely narrow 5 neV (= 5 × 10^−9^ eV) natural linewidth of the nuclear back radiation (*hν*_1_ in Figure 1a). Meanwhile, the NRVS signal (lifetime on the order of nanoseconds) can be well separated from the electronic scattering (lifetime on the order of femtoseconds) in the time domain. Thus, NRVS does not need to extract the signal from background via a low-throughput diffraction spectrometer, such as in the case of inelastic X-ray scattering (IXS) [50,51]. The time domain distinction makes NRVS spectra have higher signal throughput, an almost zero background, and with a true zero wave number (cm^−1^) start, which in turn makes the measurement on extremely weak (0.1 counts per seconds or 0.1 cts/s) signal possible, such as the Fe–H–Ni vibrational peak [7,8] in Figure 1b. A 0 cm^−1^ starting point is well illustrated in Figure 1b (as well as subsequent figures). For various reasons, IR/Raman spectra often start from 80 cm^−1^ instead. Due to the high throughput, e.g., in comparison with IXS, NRVS uses 1/10 of the incident beam flux but obtains 10 times of the signal level, which is perfect for measuring radiation-sensitive samples.

As NRVS spectra only show the vibrations associated with the isotope involved in the nuclear transition, e.g., ^57^Fe, the resulting vibrational spectrum is, thus, simpler and easier to be interpreted even without a theoretical simulation. For example, as illustrated in Figure 1b, various ^57^Fe–X bands can be identified. Fe–S is in the lower end of the spectra because Fe–S has a moderate interaction strength and S is heavier than C or H. On the other hand, Fe–H-related vibration is at an obviously higher-energy position because it has a stronger Fe–H interaction and a very light H mass. Since H has a very small mass, Fe–H vibrations will occur mainly at the H atom instead of the Fe atom, making its NRVS signal much smaller in comparison with Fe–S or Fe–CO (Figure 1b). These trends can be understood and rationalized even without theoretical simulation.

In the practical aspects, NRVS also has a number of compelling advantages over the established methods; for example, it is water-transparent in comparison with far-IR spectroscopy and, thus, well suitable for studies on biological samples in their natural environment or other samples in aqueous solution; it is free of fluorescence problems in comparison with resonance Raman spectroscopy (RR spectroscopy) and, thus, suitable for studies on photosensitive states/samples; it distinguishes well among O, N, and C ligands in comparison with X-ray crystallography or extended X-ray absorption fine structure (EXAFS). Therefore, in addition to its wide applications in physics and material sciences, NRVS is now the third modern X-ray spectroscopy technique, which has been widely welcomed by biochemical researchers, following X-ray crystallography and EXAFS.

## 2. Experimental Aspects (I): Instruments

As this article focuses on the experimental science, we use the next three major sections to discuss them. We discuss the instrumentation needed for NRVS in this section, the experimental procedures or practical issues in Section 3, and the data processing and evaluation in Section 4.

### 2.1. Detecting Inelastic Scattering

Inelastic processes, such as creation and annihilation of phonons (i.e., exciting or de-exciting lattice vibration quasi particles), are reflected in the spectra as side peaks to the large central peak coming from elastic scattering [41]. For measuring an inelastic process, researchers need to (1) define the incident energy E_1_ and measure the scattered energy E_2_ accurately in order to determine the energy transfer (E_1_–E_2_) accurately, and (2) extract the weak inelastic scattering signal from the huge background. In principle, a diffraction-based monochromator can provide and scan the incident energy E_1_ and a diffraction-based spectrometer can resolve the scattered energy E_2_. In order to measure phonons (vibrations), both E_1_ and E_2_ need to have about 1 meV (8 cm^−1^) energy resolution, such as in the case of the conventional RR spectroscopy or the synchrotron-based IXS [50]. A diffraction-based spectrometer has a low throughput because it disperses both energy and momentum. However, NRVS does not measure and thus does not care about the momentum dispersion. Meanwhile, the nuclear back transition which naturally has an extremely narrow linewidth (e.g., ~5 neV for ^57^Fe) and can act as an excellent intrinsic “spectrometer” for E_2_. Therefore, NRVS measurements do not need a diffraction-based low-throughput spectrometer to measure the scattered energy E_2_, which enables NRVS to have a much higher photon in and photon out efficiency. For example, IXS [50] uses an incident beam flux on the order of 10^10^ photons/s and produces ~1 cts/s for the asymmetric Fe–Cl stretch at 380 cm^−1^ in an [NEt_4_][FeCl_4_] sample; NRVS uses a beam flux on the order of 10^9^ photons/s and has ~10 cts/s for the same vibrational feature. In this case, with one order less photon flux, we obtain one order higher signal—an improvement by two orders of magnitude.

Although the scattered energy E_2_ can be well defined by the nuclear back transition itself, the photons collected by the detector(s) include not only the signal from the nuclear scattering event but also the unwanted background counts from the electron scattering (often on the order of 20 million cts/s in total for a four-element detector array at SPring-8). Since NRVS uses no energy dispersion spectrometer, it is necessary to resolve the signal from the background via some other mechanism, for example, in the time domain. As nuclear scattering processes have a long lifetime (on the order of ns, e.g., ^57^Fe at 14.4 keV has a 1/e lifetime of 143 ns), whereas X-ray-electron interactions are essentially instantaneous (fs), leaving the background’s pulse duration the same as the SR pulse duration (e.g., 70 ps). This enables distinguishing the nuclear scattering contribution from the electron scattering background in the time domain, thus requiring no use of a diffraction-based spectrometer. Although the SR source can provide a much higher incident beam intensity, it also brings a much higher background—the electron scattering. Therefore, the key to an SR-based NRS (including NRVS) experiment is to exploit the nanosecond (ns) time delay for the nuclear event and exclude the much faster (fs) electron scattering events. This is similar to the case of laser-induced fluorescence (LIF) spectroscopy [52–55].

### 2.2. Light Sources for NRVS

Any spectroscopy needs a stimulating impulse (such as a light source), a sample, and a detecting system. A spectroscopic light source can be as simple as a light bulb (UV–visible spectroscopy) or as complicated as a laser system (RR spectroscopy) or even SR source (NRVS, IXS).

There are more than 60 operational SR storage rings worldwide. However, not all of them are suitable for hosting an NRS beamline or performing a NRVS experiment. There are some *conditio sine qua non* requirements for the light source for a NRVS experiment.

These requirements include (1) a narrow bandwidth for the incident X-ray, on the order of 1 meV or less, (2) a high enough beam intensity to create a 1 meV bandwidth X-ray but still with a flux on the order of 10^9^ photons/s or higher, and (3) a time structure with its pulse interval matching or being greater than the isotope’s lifetime scale, e.g., >143 ns for ^57^Fe. Although requirement (1) demands specialized X-ray optics at an NRS beamline, the remaining requirements depend on the type of SR rings. A large circumference ring is required to facilitate high electron storage energy and low emittance, which are important for a NRVS experiment. In addition, an ^57^Fe NRVS prefers an SR ring to have a large enough circumference to allow a wide enough space interval for a long enough electron bunch interval and to have large enough numbers of electron bunches at the same time (in order to have a sufficiently high beam flux and, accordingly, stable beam quality). A simple mathematics tells us that the 143 ns time interval corresponds to a 42.9 m space interval between the electron bunches inside the SR ring. Therefore, a ring with a sizable circumference (about or over 1 km) is also preferable in order to have a sufficient number of electron bunches.

**Figure 3.**
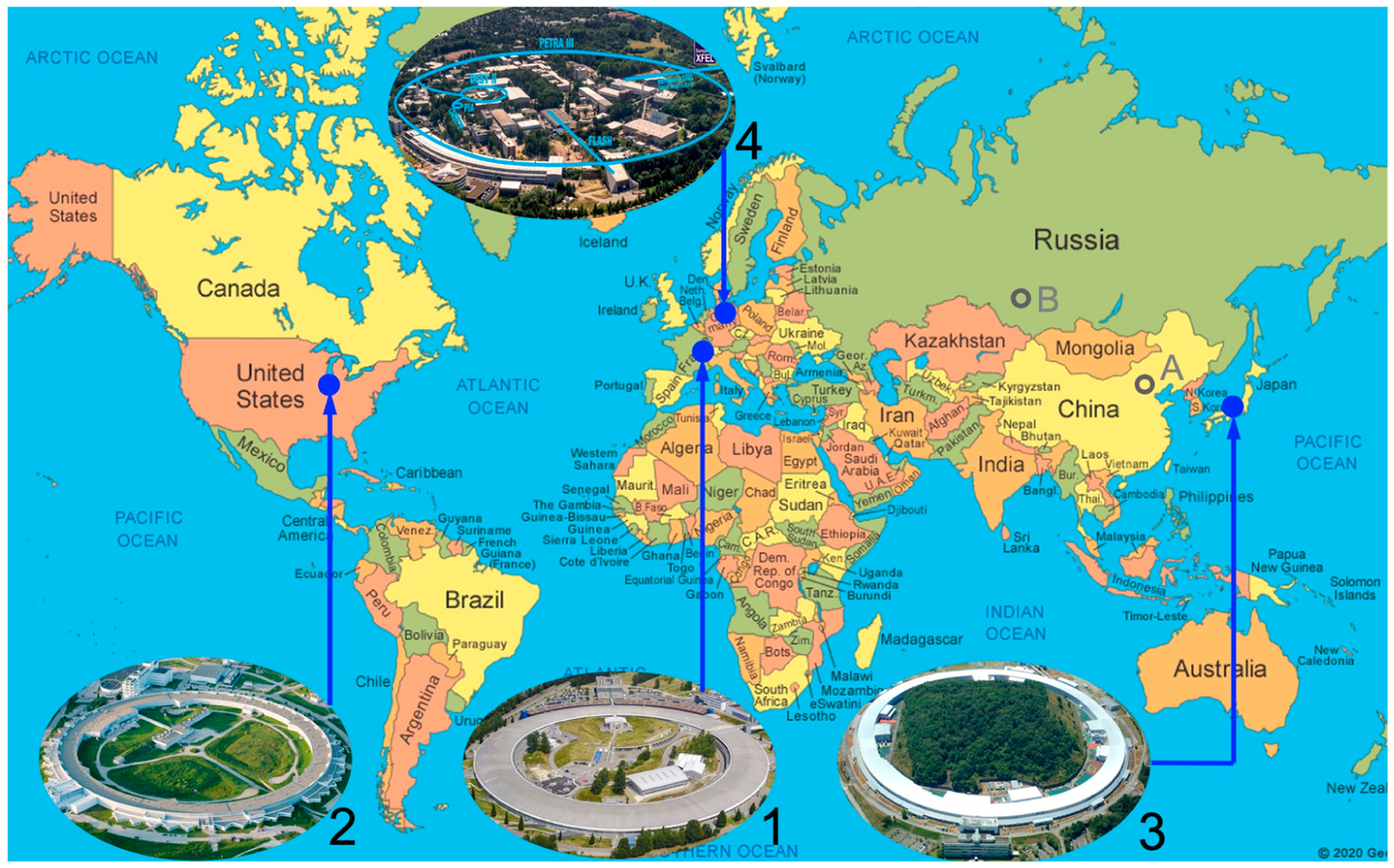
Synchrotron radiation facilities around the world which have nuclear resonant scattering (NRS) beamlines (solid blue symbols): 1 = ESRF, with 6 GeV electron energy and 0.9 km circumference, operating since 1994; 2 = APS, with 7 GeV electron energy and 1.1 km circumference, operating since 1995; 3 = SPring-8, with 8 GeV electron energy and 1.5 km circumference, operating since 1996; 4 = Petra–III, with 6 GeV electron energy and 2.3 km circumference, operating since 2009. The gray circles indicate the possible NRVS-suitable rings in the future: A = HEPS, with 6 GeV electron energy and 1.4 km circumference; SKIF, with 3 GeV electron energy and 0.5 km circumference.

A high-energy third-generation SR has a large circumference and a pulsed time structure and, thus, is suitable for NRS and NRVS experiments. Currently, there are only four SR rings in the world that meet these requirements and allow NRS or NRVS experiments. These “super” SR rings are shown as in Figure 3: ESRF in Grenoble, France (1), with 6 GeV electron energy and 0.9 km circumference, operating since 1994; APS in Chicago, USA (2), with 7 GeV electron energy and 1.1 km circumference, operating since 1995; SPring-8 in Hyogo, Japan (3), with 8 GeV electron energy and 1.5 km circumference, operating since 1996; Petra-III in Hamburg, Germany (4), with 6 GeV electron energy and 2.3 km circumference, operating since 2009. At APS, there is an operation mode with 24 electron bunches or (1100 m)/(0.3 m/ns)/24 = 153 ns time interval for experiments on ^57^Fe. At SPring-8, C mode has 29 electron clusters or (1450 m)/(0.3 m/ns)/29 = 165.2 ns period. For NRVS, there can be more than one electron bunch within each electron cluster; SPring–8 has 11 bunches in each C mode cluster and the real bunch interval is 145.5 ns instead of 165.2 ns. For other isotopes, there are other operational modes with different time intervals available; some of them are over 1 μs. There are several NRS beamlines in each of these super SR rings. For example, APS has 03ID, 16ID, and 30ID, while SPring-8 has BL09XU, BL10XU, BL11XU, BL35XU, and BL19LXU (mobile NRS), ESRF has ID18, Petra-III has P01, etc.

More NRS-compatible SR rings are planned or under construction around the world, including HEPS in China (A, 6 GeV, 1.4 km) and SKIF in Russia (3 GeV, 0.5 km). The interesting fact about SKIF is that, although it has a 3 GeV ring, it will use superconducting undulators to boost photon flux and make it suitable for NRVS, with a 75 ns time interval, a 2 meV energy resolution, and ~10^10^ photons/s flux. While such an alternative approach may or may not be a good idea, it at least shows researchers’ enthusiasm to pursue NRS or NRVS experiments.

A third-generation high-energy SR ring in general has higher radiation output. In addition, the larger circumference also leads to a larger space for each beamline, which makes it possible to use a longer undulator to provide even higher beam flux, such as the 25 m long undulator used at SPring-8 BL19LXU [56]. Different NRS beamlines have different advantages. For a smooth start, initial NRVS users are encouraged to first contact the corresponding beamline scientist(s) prior to their NRVS research and/or proposals.

### 2.3. High-Resolution Monochromators

**Figure 4.**
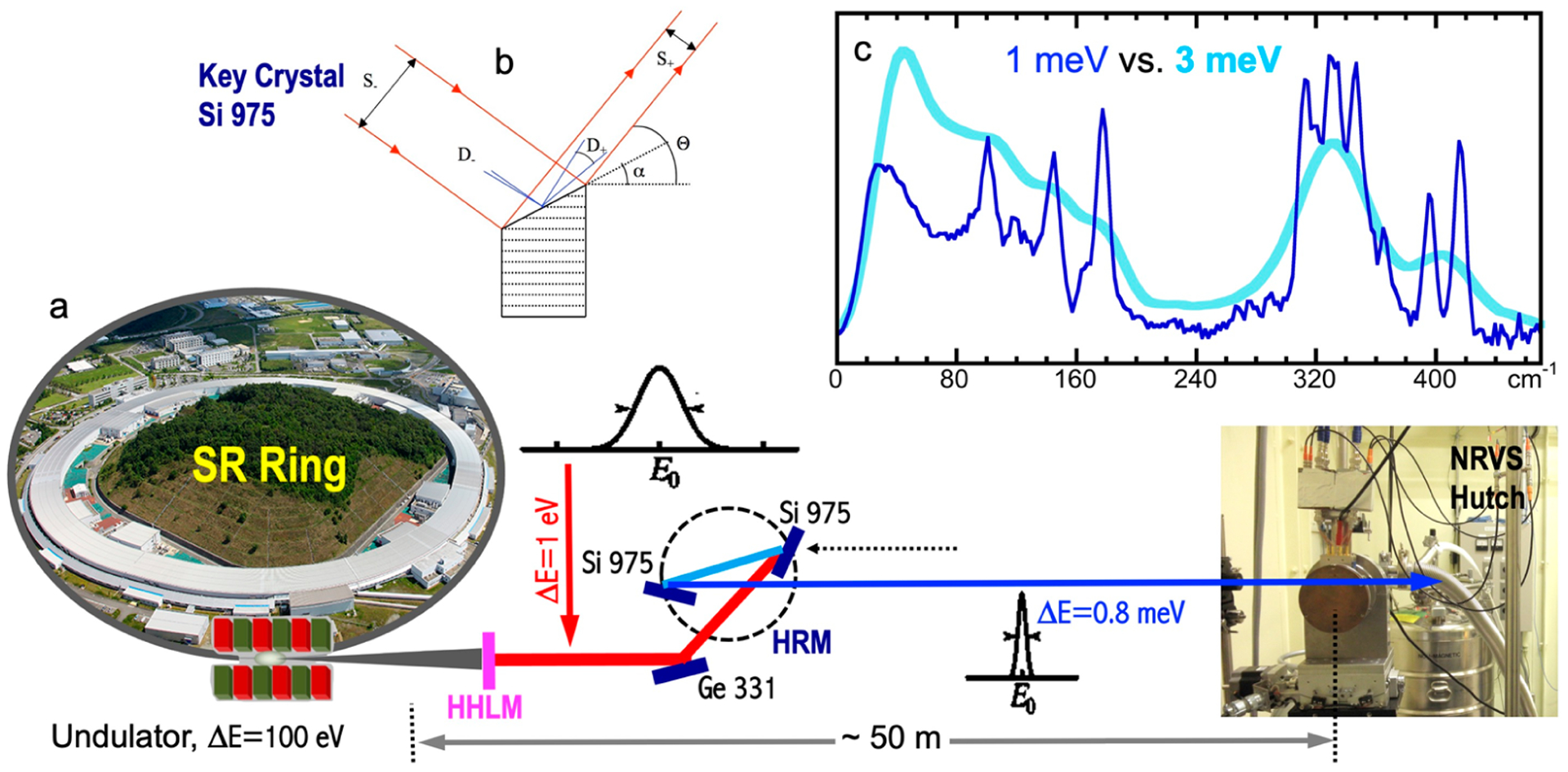
A schematic illustration from an SR ring to NRVS beamline to measurement hutch (**a**), along with an illustration for an asymmetrically cut monochromator crystal and its diffraction characteristics (**b**), and the NRVS spectra measured for [Fe_2_S_2_Cl_4_]^=^ with a 1 meV (thin dark blue) vs. a 3 meV (thick light blue) HRM (**c**).

In a third generation SR storage ring, while bending magnets make the electrons travel in a circle, it is various insertion devices that emit the X-ray beam. The complex magnetic force causes an electron beam to perform wiggles (in a wiggler) or undulations (in an undulator) as they pass through the devices. Through an undulator, the radiation constructively interferes with the motion of the oscillating electrons, resulting in a beam of radiation with a partial coherence. In addition to creating a brighter light, an undulator also enables tuning of the light so that different central energy can be generated for different applications by varying the “undulator gap” between the magnets. As illustrated in Figure 4, the “white X-ray” that comes out of an undulator has a narrow angular divergence, a high brightness, and 100 eV in energy distribution width (dark gray). At a beamline, it will first be reduced to ~1 eV in bandwidth with a high-heat-load monochromator (HHLM, vertical pink in Figure 4), which is good for the vast majority of X-ray spectroscopic work, such as EXAFS [57].

In order to measure NRVS, it is essential to have an energy resolution at ~1 meV (=8 cm^−1^), along with a scanning span of ~100 meV [58,59]. This requires a second monochromator, a high-resolution monochromator (HRM, dark blue), which is the most important X-ray optics device at an NRS beamline. The HRMs at BL09XU and BL19LXU at SPring-8 reach an energy resolution of 0.8 meV for ^57^Fe experiments. In general, a resolution of 1.0–0.2 meV can be achieved according to users’ need and depending on the nuclear transitions targeted. For example, a 0.27 meV HRM is available at APS 03ID for ^151^Eu NRS experiments [60] and is, thus, of potential interest for researchers who study europium and its photoluminescence (see, for example, [61,62]). On the other hand, HRMs with 2–3 meV resolution and a higher beam flux also exist and can be used in the measurements of special features. Once an NRS beamline is built, the undulator and HHLM are tuned to provide an intense peak at around the nuclear resonant energy, e.g., 14.4 keV for ^57^Fe. During the HRM scanning over an energy span of ±100 meV, there is no need to track the undulator or HHLM position as the beam resolution at HHLM is 1 eV, which is much wider than the scanning range at HRM (±0.1 eV) [2,63].

According to Bragg’s law,

(3) 2dsinθ=nλ

where n is the order of diffraction, d is the spacing between diffraction crystal planes, *λ* is the wavelength of energy being diffracted, and *θ* is the scattering angle (diffraction angle); different energies are, thus, diffracted onto different directions, which is the basis for designing a monochromator of any kind. Regular diffraction refers to the case in which the incident and the diffracted beams are symmetric with respect to the crystal’s cut surface, for example, the Si(1,1,1) crystal used in a HHLM. On the other hand, HRM uses an asymmetrical diffraction, which means that the cut surface of the crystal is not in parallel with the diffraction crystal plane and, therefore, the incident and diffracted X-rays are not symmetric with respect to the crystal’s cut surface; of course, they will always be symmetric with respect to the diffraction plane. An asymmetric diffraction can realize a larger beam dispersion angle (D^−^ → D^+^), while it has a narrower beam size (S^−^ → S^+^), as illustrated in Figure 4b, which is suitable for an HRM. To further disperse the beams with different energies, the normal angles of these two diffraction crystal planes are arranged between 90° and 180°, forming a ++ (dispersive) crystal array. The paired asymmetric crystals are referred to as the key diffractive crystals. For example, the HRM at SPring-8 BL09XU or BL19LXU has a paired Si(9,7,5) crystals as the HRM’s key diffraction crystals. The angle variation between the two key diffraction crystals defines the exact value of the output energy.

Since the range of the angle scan between two key diffraction crystals is pretty small while its accuracy needs to be fairly high, its tuning is performed via a piezoelectric actuator rather than via a stepper motor. Unlike stepper motors, piezoelectric devices do not have a mechanical backlash. Meanwhile, the rotation of the whole key crystal pair unit, as well as other mirrors, is tuned by stepper motors to optimize the beam flux and/or to manipulate the beam position onto the sample position. Due to the high dispersion, as well as the high index diffraction, the produced ~1 meV X-ray beam has a flux on the order of as small as only 10^−9^ photons/s centered at 14.4 keV, four orders lower than the beam with 1 eV resolution after the HHLM. The measured NRVS signal is often on the order of 0.1–1 cts/s, which is rather weak in comparison with other X-ray spectroscopies (e.g., EXAFS). This is another reason why a super SR source (Figure 3) has to be used for an NRVS experiment.

The necessity of using a ~1 meV HRM in NRVS experiments is demonstrated in Figure 4c with NRVS spectra for [Fe_2_S_2_Cl_4_]^=^ measured with a 1 meV (thin dark blue) vs. a 3 meV (thick light blue) HRM. While the 1 meV spectrum has rich features of resolved vibrational mode(s), the 3 meV spectrum only shows two big bumps. On the other hand, although HRMs much better than 1 meV exist, they will not provide much more information except for a few special cases, because most vibrational transitions in chemical complexes or in enzymes have more than 8 cm^−1^ (1 meV) linewidth. This makes ~1 meV HRM one of the key elements for a successful NRVS measurement.

There are three-crystal HRMs and four-crystal HRMs. Using ^57^Fe HRM as an example, a three-crystal HRM is installed at the SPring-8 BL09XU (and also used at BL19LXU). It has a pair of Si (9,7,5) key crystals to tune the 0.8 meV resolution beam, while a Ge (3,3,1) crystal is used as a front mirror to adjust the input beam direction, as shown in Figure 4a. At APS 03ID, a four-crystal HRM is used with a pair of Si (10,6,4) as its key crystals, along with two Si (4,0,0)s being at the front and the back as light path manipulators. At the latest built Petra–III P01, a structure of 2Si (10,6,4) × 2Si (4,0,0) is also used for its HRM. A HRM usually produces a beam with 1 × 0.6 mm^2^ in cross-section size. Some beamlines have additional Kirkpatrick–Baez (KB) focusing mirrors [64] to further focus the beam to a cross section of 0.1 × 0.1 mm^2^ or even smaller. Dedicated NRS beamlines often have separate hutches for HRM and for nuclear measurements to minimize the influence of human activities on HRM’s exit energies.

Mobile HRMs that can be built at a nondedicated NRS beamline for temporary use are an important development direction for NRVS or NRS experiments. For example, there is no HRM installed at BL19LXU of SPring-8; thus, one could not measure NRVS there. However, it has a 25 m long undulator and severalfold greater incident beam flux [56] in comparison with other beamlines, allowing people to measure weaker signals. Yoda and his research team built a mobile HRM and a mobile nuclear measurement instrument that can let researchers use this excellent beamline for NRVS measurements, paving the way to extend NRVS or other nuclear experiments to non-NRS beamlines. People need 36–48 h to move in and set up the HRM and other instruments at the beginning of each beamtime [8]. Nevertheless, researchers are rewarded with amazing NRVS results [7,8,45,47,65–69].

### 2.4. X-ray Interaction with NRVS Sample

**Figure 5.**
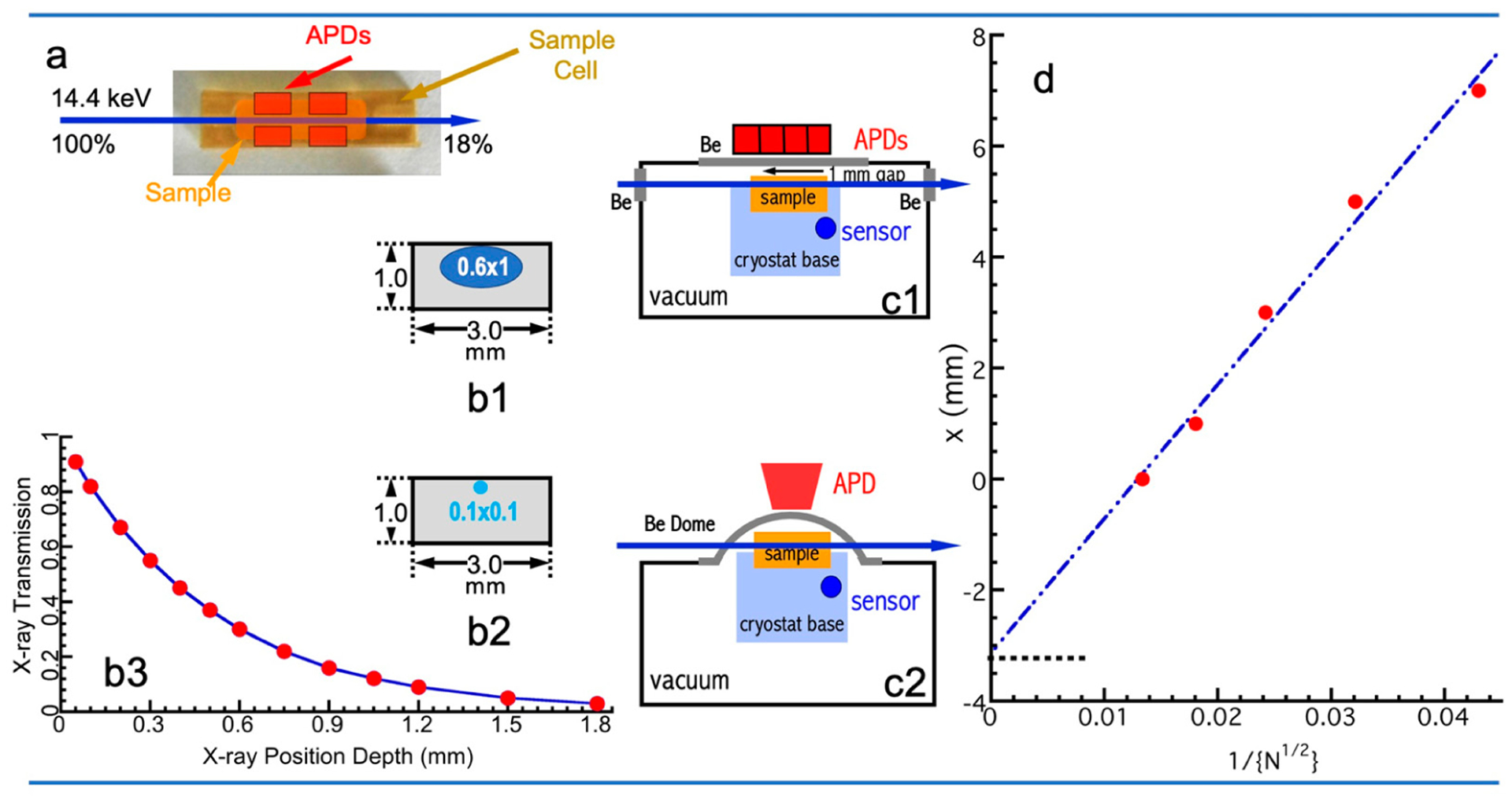
(**a**) Geometric relationship among the >10 mm long sample/sample cell, incident X-ray beam, and APD detector array from the top view; (**b**) the relationship between X-ray beam and sample from the front view (cross-section) with a beam size of 1 × 0.6 mm^2^ (**b1**) and 0.1 × 0.1 mm^2^ (**b2**); (**b3**) the 6.4 keV X-ray transmission rate at different depth under an H_2_O sample; (**c**) side view of the incident beam, sample, and APDs: (**c1**) with a flat beryllium window in cryostat between the sample and the APDs; (**c2**) with a beryllium dome in cryostat; (**d**) the linear fit to the data of nominal sample-APD distance (x) vs. 1/(N^1/2^), where N is the nuclear resonant signal.

To better describe the geometric relationship among the sample, the incident beam, and the location of the detector(s), we define the following view positions: the view from the incident beam is called front view; the view from the detector(s) is called top view, although detector(s) can be on top, side, or even bottom of the NRVS sample; the view from the direction perpendicular to both beam and sample-detector(s)’ direction is defined as side view. The top view of the beam, sample, and detectors along the direction of beam propagation is shown in Figure 5a, while the front view which shows the cross-section of the sample-beam relation is shown in (b1) assuming a beam size of 1 × 0.6 mm^2^ and in (b2) assuming a small beam size of 0.1 × 0.1 mm^2^. To optimize the 14.4 keV X-ray interaction with the sample, an aqueous solution sample should have a pretty long longitudinal length (Figure 5a). A simple calculation shows that a 10 mm long H_2_O sample (to simulate a biological sample) can achieve over 82% beam–sample interaction (18% transmission). Meanwhile, the sample should also be longer than the dimension covered by the detectors to ensure sufficient detection.

On the other hand, the escape depth for 90% of the NRVS signal, i.e., the internally converted Fe K_α_ X-ray fluorescence at hν_2_ (= 6.4 keV), is much smaller than 10 mm. Figure 5b3 illustrates the relationship between the escape rate of the Fe K_α_ fluorescence and the beam depth in the NRVS sample. If the beam center is at the depth of 0.3 mm, the escape rate is approximately 54% at that position. The closer the beam center is to the surface of the sample, the higher the escaped rate of the X-ray fluorescence and, thus, the higher the NRVS signal will be; however, there will be smaller overlap between the beam and the sample, meaning less sample-beam interaction and, thus, a lower level for the final NRVS signal. In this sense, a smaller beam size (e.g., 0.1 × 0.1 mm^2^) will have a better NRVS signal than a larger beam size (e.g., 1 × 0.6 mm^2^). According to Figure 5b3, (b1) will have a beam escape rate of 54% (beam centered at 0.3 mm depth), while (b2) can have a beam escape rate of 92% (beam centered at 0.05 mm depth). The grazing incidence angle of the beam relative to the sample should also be optimized around 0°–6°. In practice, a solution sample cell has a cuvette size of 10 × 3.5 × 1 = 35 mm^3^, while a sample cell for powder samples has a smaller cuvette because powder samples have obviously higher attenuation for X-rays than H_2_O.

A cryogenic temperature improves the samples’ LM factor, enhances the single-phonon contribution to the spectrum, makes vibrational spectral lines sharper due to the reduction in dynamic sample disorder, and ensures more particles in the vibrational ground state. Therefore, most NRVS samples (even nonbiological samples) are measured at an as-low-as-possible temperature, although some NRVS samples do have a noticeable radiation damage. Since NRVS uses a much lower photon flux, the sample-detector position should be as close as possible to enhance the detection solid angle, and a cold-finger cryostat (instead of a helium gas heat exchange cryostat such as the one in an EXAFS experiment [57]) has to be used for this purpose. Figure 5c1,c2 show the side view for the beam–sample-detector relation from side view. A beryllium window is used between the sample (at cryogenic temperature) and the detector (at RT). There are two types of beryllium windows—flat ones (c1) and dome-shaped ones (c2). The former allows an extremely short sample-detector distance (such as ~3 mm used at SPring-8 BL09XU), while the latter allows the detection angle to be freely adjusted (such as the one used at APS 03ID).

Although visual and geometric measurements can provide an estimate, the real sample-detector distance includes the sample’s bumpiness, the vacuum window thickness for both cryostat chamber and detector, and the air gaps in both the cryostat chamber and the detector unit; therefore, it is difficult to be measured with accuracy. Since the detector area is fixed, the nuclear resonant signal (N) should be inversely proportional to the square of the real sample-detector distance (d + x)^2^; then,

(4) $$\text{x}=\text{k}\cdot (1/\sqrt{\text{N}})-\text{d}$$

where d is the “minimum distance”, x is the added distance, and k is a constant. People can then back up the detector(s) from the “minimum distance” (d) to a series of locations with added distance (x_i_) and measure the corresponding nuclear resonant signal (N_i_). A linear least square fit to the x_i_ vs. 1/(N_i_^1/2^) will lead to the values for k and d, e.g., d = − 3.2 mm in Figure 5d. This is an accurate method to define the distance between the sample (from the beam-sample interaction center) and the detectors (to the detectors’ elements inside the vacuum). We recommend each NRS beamline publish or provide these data for NRVS users to understand the real sample–detector distance.

### 2.5. Detectors

Although the recoil fraction may have an overall cross-section comparable to the recoilless fraction in a nuclear resonant scattering, the intensity of the recoiled spectrum is spread over tens or hundreds of meV, compared to tens of neV for nuclear resonant scattering (Mössbauer spectroscopy). Thus, the absorption cross-section at each particular peak is effectively six orders of magnitude weaker than that from Mössbauer spectroscopy. Therefore, the NRVS detector must have a good detection sensitivity. In order to distinguish the NRVS signal from the huge but fast electronic scattering background, the corresponding detector must also have a fast time resolution and a super saturation level. Avalanche photodiode detectors (APDs) possess these properties and, thus, are suitable for measuring nuclear events, including NRVS [70,71].

While “normal” PIN photodiodes work with a positive bias and a “normal” gain, an APD works with a reversed bias near its breakdown voltage, e.g., a negative few hundred volts for Si. There are two working conditions for APDs. One condition is to operate carefully with a reversed bias close to but not quite at the breakdown voltage, which leads to thousandfold improved signal gain. Another condition is to operate with a reversed bias at or even a bit over the breakdown voltage but with an additional mechanism to control the reverse current so that the device will not burn. The latter option can lead to an extremely high gain (millionfold improvement), providing excellent detection sensitivity. APDs also have an extremely low dark current on the order of 0.01 cts/s and an extremely high saturation level. It is about 6MHz per element, no matter how large the element area is. Therefore, using an APD array with small individual elements instead of a single APD with large area is advantageous. The combined high gain, low dark current, and high saturation rate make APDs suitable for measuring NRVS, which has a weak signal to extract but a huge background to handle with at the same time.

In addition, APDs also have a fast counting and fast processing rate and, thus, a high time resolution, which is critical for distinguishing the delayed nuclear events (e.g., NRVS) from the huge but fast electron scattering background. Modern APDs can have a time resolution of 1 ns or better.

### 2.6. Data-Acquisition Electronics

**Figure 6.**
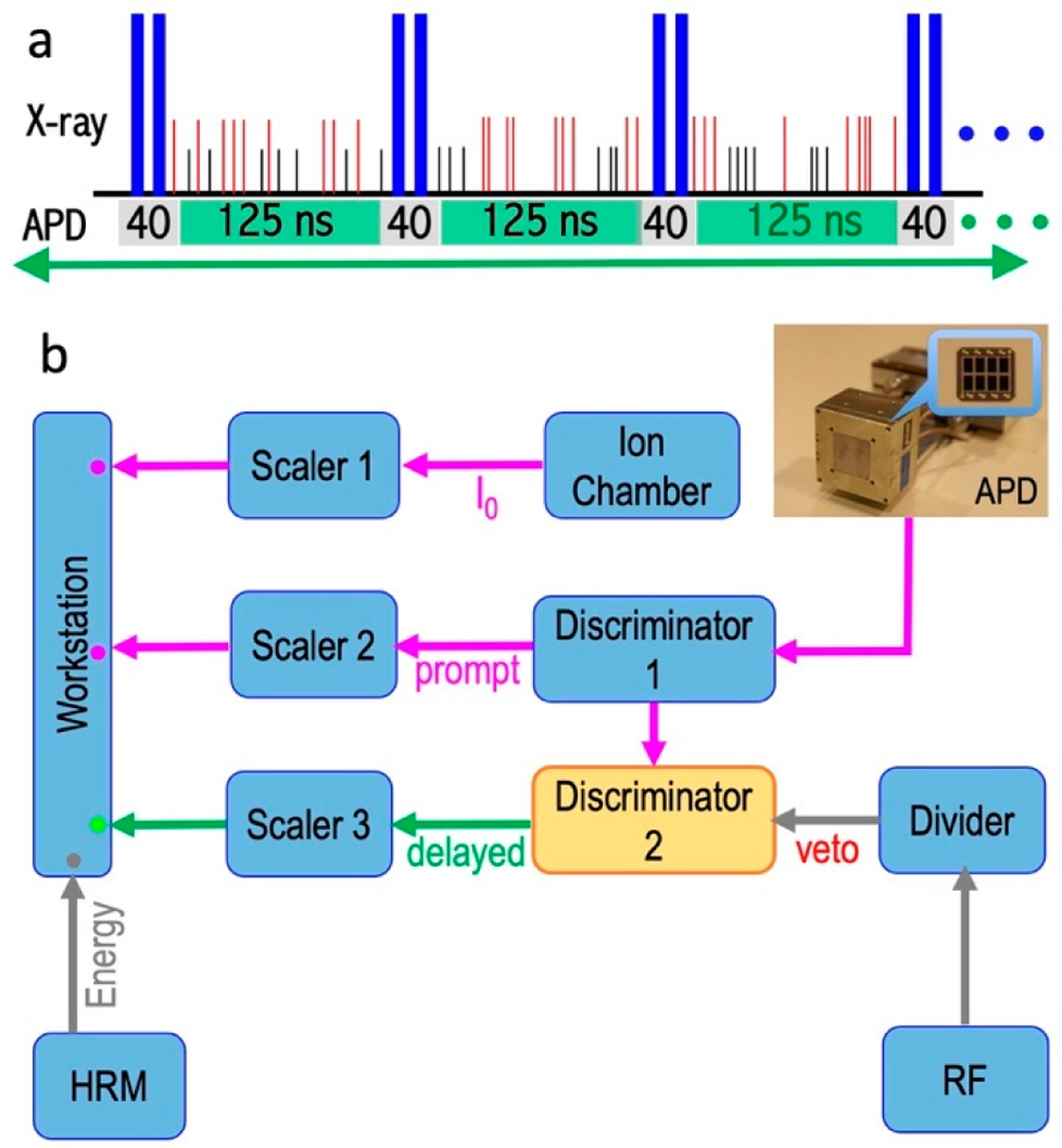
(**a**) A typical timing scheme for an NRVS experiment. No count is collected during the 40 ns period (gray) around the incoming X-ray pulses, while the counts are being collected as the NRVS signals in the remaining 125 ns period (green, no veto period). This timing is based on SPring-8 C-mode operational conditions; (**b**) nuclear electronics used to separate the delayed nuclear events from prompt electron scattering events. The top right insert is a photo for an APD array used at SPring-8 BL09XU. The purple lines represent the X-ray signals without time structure or those with mixed time structures; green lines stand for the delayed nuclear signals; grey lines are for the electric signals, such as HRM positions or timing.

APDs collect the time-resolved photon signals, but it is the data-acquisition electronics which (also called nuclear electronics) finally separate the slow nuclear scattering signal from the huge and fast electronic scattering background in the time domain. The X-ray beam pulses emitted at the beamline correspond to the electron bunches traveled in the ring. Using the C mode at SPring-8 as an example, as the 11 electron bunches (in one cluster) pass through the undulator, a series of 11 X-ray pulses are emitted. Although each pulse has a time width of 40 ps, the series of 11 incoming beam pulses are spread in a time block (duration) of 19.7 ns, with a block period of 165.2 ns and a time interval of 145.5 ns between different time blocks, as mentioned in Section 2.2. Figure 6a illustrates an intuitive time spectrum for an NRS (including NRVS) process; there are nuclear scattering signals (the thin red and black vertical bars) following one block of 11 incoming beam pulses (represented with pairs of thick dark blue bars) and prior to the next block. The NRVS system needs to be “off” from collecting counts in about 40 ns (gray), which covers the whole 19.7 ns (~ 20 ns) time block and −4/+16 ns at each end of the time block. The electronics will collect the delayed nuclear scattering counts in the remaining 125 ns (green).

To understand how to do this electronically, a block diagram for the data-acquisition electronics used at SPring-8 BL09XU is shown in Figure 6b. The first important device is a divider which acts as a “bunch clock,” and tells the electronics the initial time point of t = 0 according to the arrival of the RF signal (e.g., the first thick blue bar in each cycle in Figure 6a). The most critical devices are fast timing discriminators (or discriminators for short). The Discriminator 1 is following the APDs to fast process the timing information of the collected pulses. The most important data-acquisition step is the co-process of the APDs collected (and Discriminator 1 processed) counts and the divider-issued triggers meet in the second discriminator (Discriminator 2 in Figure 6b). When the Discriminator 1 send APD collected signals to the Discriminator 2 during the “no veto” period, the signals will past through Discriminator 2 to the Scaler 3, at which the signals will then be digitized and sent to the workstation (green dot in Figure 6b). In practice, a veto interval (e.g., from −14 to +26 ns here) is set to cover the duration of the synchrotron pulse block (with an additional −4/+16 ns margin at each end), to avoid the signal integration during the period with possible electron scattering and to allow the electronics to have time to recover. On the other hand, the counts that occur in the “on” times (no veto, e.g., t = 26–151 ns) are passing through the Discriminator 2 and digitized as final NRVS counts. The values without time structure, e.g., I_0_, prompts, and energy positions are directly digitized to send to the workstation for storage (the dots in purple or grey). Such collected delayed nuclear scattering counts vs. the vibrational energy (E_1_–E_2_) form the raw NRVS spectral scans.

Pulsed signals from nuclear scattering events have random heights, random timing, and random duration. Unlike the communication-based TTL signals, the timing information for these random and irregular pulses is, thus, difficult to track. A discriminator enable: 1) the counting of narrow pulses at very high counting rates; 2) precisely recording the arrival time for these pulses; 3) separate signals in the time domain with the external triggers. They are designed to achieve a fast time resolution and the highest counting rates by operating on the fast-rising detector signal. Although the detailed nuclear electronics theory (or the associated design) is out of the scope of this article, it is not hard for a user to understand that discriminators are suitable to work with APDs and thus are good for NRVS measurements. Or, in another word, discriminator is one of the most important components in NRVS data-acquisition electronics.

## 3. Experimental Aspects (II): Measurements

Once samples are prepared and delivered to the NRS beamline, the general experimental procedure incudes loading the sample into the sample base inside the cryostat, evacuating the cryostat chamber, cooling down the sample to an as-low-as-possible temperature (e.g., 10 K in the temperature senor) or other designated temperature, placing the APD detector next to the beryllium window of the vacuum chamber, closing the hutch, adding bias voltage to the APDs, and starting the NRVS measurement.

The initial measurement also includes finding/optimizing the nuclear resonant peak, adjusting the sample’s position to maximize the nuclear resonant signal, checking for the dark count rate, and performing energy calibration.

### 3.1. Checking for Dark Count Rate

An important procedure for NRVS measurement preparation is to find the NRVS background count rate (also called the dark count rate, or dark current). This must be done with (any) sample loaded, in the same timing window (in the electronics setting) for a regular NRVS measurement and at the energy position of −200 meV where the real NRVS signal should be zero.

The dark counts are not only from APD detectors. The total dark count rate for an NRVS measurement includes (1) the real electronic dark current from the detector (and data-acquisition electronics), (2) minor irregularities in X-ray pulses due to the minor irregularity for the electron bunches in the SR ring, and (3) the background cosmic ray radiation. In most cases, setting a narrower time region is a solution to control the dark counts from (2) if these counts are outside but close to the block of normal electron bunches. In some cases, a second window can be set to eliminate the noise counts in certain time region. Ultimately, an improvement of SR operation is needed to control the noise source from (2). In addition to the timing window, one can set a rough lower energy threshold in the second discriminator (Figure 6b) to discriminate against electronic noise (1) and a rough upper energy limit to reject cosmic ray photons (3).

With all the suitable conditions met, a good system should have a background count rate of less than 0.05 cts/s. For example, the one at SPring-8 BL09XU or BL19LXU is about 0.03 cts/s on average. As a convention, the background count rate check can be done with five 100 s count accumulations, or with a single 500 s accumulation. An extremely low background count is a crucial factor in setting the lower detection limit for the NRVS signals. If the background count rate appears to be more than 0.05/s, the timing window in data-acquisition electronics is among the first items to address. Sometimes, in addition to other methods, the APD bias must be reduced to compromise the gain but simultaneously reduce the unwanted background counts. The electronic windows are set by the scientist operating the beamline. However, it is important for an NRVS user to understand the basics, the available options, and their impact on and importance to the success of a critical experiment, especially for measuring weak features and/or dilute samples.

### 3.2. Energy Calibration

**Figure 7.**
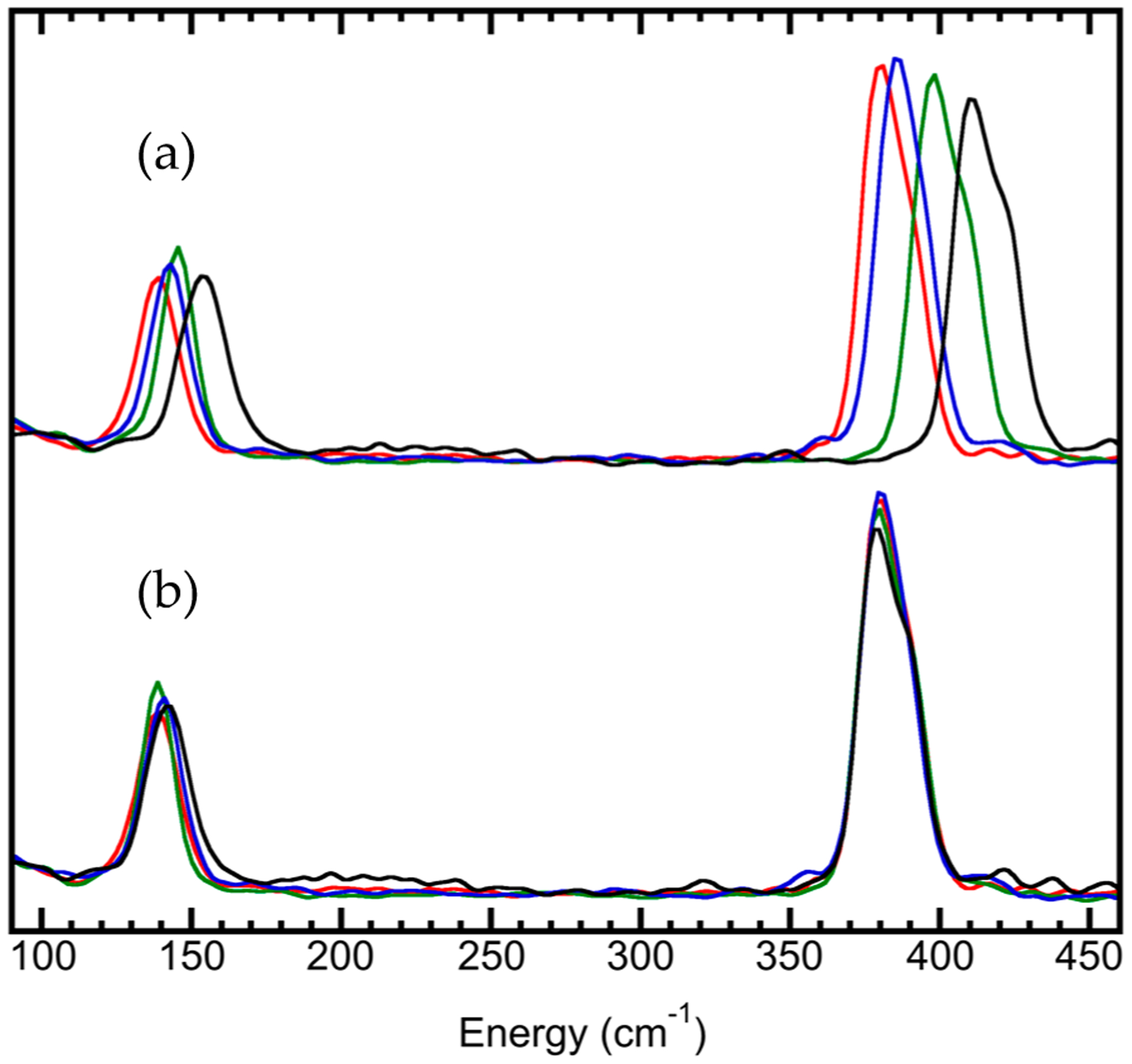
The uncalibrated (**a**) and calibrated (**b**) NRVS spectra for [NEt_4_][FeCl_4_] measured at four beamlines at APS 03ID (red), energy scale = 0.999; ESRF ID18 (blue), energy scale = 0.986; SPring-8 BL09XU (green), energy scale = 0.965; and Petra–III P01 (black), energy scale = 0.920.

Like for other spectroscopies [72,73], preparation for an NRVS measurement must include the beam energy calibration [59]. The nuclear resonant position E_0_ can be automatically aligned during the data analysis in the software while the energy axis scale is often carried out with a calibration sample with a known and prominent vibrational peak. The Fe–Cl stretch peak from the [NEt_4_][FeCl_4_] complex is one of them. Its NRVS values measured at different beamlines (Figure 7a) do not overlap with each other; the difference can be up to 4 meV (or 32 cm^−1^) from each other. However, a linear correction factor (called the energy axis scale) of about 0.920–0.999 can bring all of them (Figure 7a) into a very good alignment with the reported Fe–Cl position at 380 cm^−1^ (Figure 7b) (as well as its IR peak at 380 cm^−1^) [48]. In addition, the NRVS spectrum for (NH_4_)_2_MgFe(CN)_6_ measured at RT can also be used as an alternative energy “calibrant” with a prominent peak at 602 cm^−1^ (not shown here) [16].

The primary source of energy uncertainty in the monochromator comes from changes in the crystal planes’ atomic spacing (d) due to temperature change as a result of exposure to X-rays (or changes in ambient conditions). For Si, the coefficient of thermal expansion is 2.56 × 10^−6^ K^−1^, leading to an energy shift of 9 cm^−1^ (or over 1 meV) per 0.03 K of temperature change. Although, these environmental variables are usually controlled well at an SR facility, inadvisably entering the monochromator hutch could change the crystal temperature by as much as 0.1%. This could lead to an energy shift in several meV and require hours to re-equilibrate. Therefore, beamline energies must be checked and even frequently checked, especially when the temperature of the HRM hutch is disturbed. All the dedicated NRS beamlines have HRM and NRVS measurement separated in different hutches to prevent the temperature surge around HRMs due to beam on and off activities. A positive aspect is that the temperature sensitivity can sometimes be intentionally used as a mechanism of tuning monochromator energy instead of rotating *θ*; for example, the backscattering HRM for IXS experiments at Spring-8 BL35XU uses such a mechanism to scan Si(11, 11, 11) around 21.8 keV with ~1.5 meV resolution [50,51]. However, this alternative type of HRM is not used for NRVS.

The energy calibration becomes more critical for NRVS measurements on dilute samples as their data-acquisition time can be very long (24–48 h), and both the zero position and the energy scale can drift more than expected. As sample change requires time (>1 h), traditional energy calibration interrupts the regular NRVS measurements. To resolve this issue, the calibration can also be done via measuring ^57^Fe powder at 285 cm^−1^ or (NH_4_)_2_MgFe(CN)_6_ at 602 cm^−1^ at RT and, thus, at a second stage outside the cryostat. Such an energy calibration measurement at RT can be a quickly started by letting the full beam go through the cryostat chamber, and it can be quickly switched back to the regular NRVS measurement afterward [59]. In case of need, even an in situ calibration can be performed at the second stage while the regular sample is measured at the cryostat stage. However, in situ calibration often has very low flux at the second stage, and it also increases the mechanical dead time (and, thus, the overall measurement time).

### 3.3. Signal-to-Noise Ratio and Detection Limit

The signal-to-noise ratio (S/N) and the lowest detection limit are a concern for all spectroscopies [72], including NRVS. Let us first evaluate an example which has a strong NRVS signal, such as the Fe–Cl stretch in the [FeCl_4_]^−^ ion (10 cts/s). In this case, a 10 s measurement will lead to 100 total counts in the Fe–Cl signal but accumulate almost no dark counts (0.03 × 10 = 0.3 cts/s, assuming that the dark count rate is 0.03 cts/s). Under this condition, we can just consider the pure statistical noise, which is roughly $\text{N}=\sqrt{\text{S}}$ and $\text{S}/\text{N}=\text{S}/\sqrt{\text{S}}=\sqrt{\text{S}}=100/10=10$, where S is the measured signal count. On the other hand, for an extremely weak peak (for example, the Ni–Fe–H wag mode in Figure 1b, 0.1 cts/s), the background rate determines the best possible S/N an experimenter can ever achieve (e.g., S/N = 0.1/0.03 = 3.3).

For dilute samples like hydrogenase enzymes, we often need to use sectional scans, meaning that a much longer data-acquisition time is used in the particular regions of interest (ROIs), which often have a weak or an extremely weak feature. For example, the overall scanning parameters we utilized for measuring NRVS in Figure 1b are as follows: 1 s/p (seconds per point) in the Fe–S region, which has an average count rate of 8 cts/s, 5 s/p for the Fe–CO/CN region (0.7 cts/s), and 30 s/p for the Ni–H–Fe wag region (0.1 cts/s). After obtaining 30 such scans, we would obtain the following: S = 8 × 1 × 30 = 240 total counts and $\text{S}/\text{N}=\sqrt{240}=15.5$ for the Fe–S features; S = 0.7 × 5 × 30 = 105 total counts and $\text{S}/\text{N}=\sqrt{105}=10.3$ for the Fe–CO features. Obviously, the S/N for Fe–S (15.5) and Fe–CO/CN (10.3) can be calculated by statistical error; for example, if we use both statistical error and instrumental error to calculate the S/N for Fe–CO, it is 105/(105 + (0.03 × 5 × 30)^2^)s^1/2^ = 10.2 instead of 10.3. For the Ni–H–Fe wag region (0.1 cts/s), however, it is important to include both instrumental dark count cts/s and statistical error in the S/N calculation in order to get a real S/N. After 30 above scans, it has S = 0.1 × 30 × 30 = 90 total counts and S/N = 90/(90 + (0.03 × 30 × 30)^2^}^1/2^ = 90/28.6 = 3.14. For a more general case, if a total data-acquisition time t is spent, S/N = 0.1t/(0.1t + (0.03t)^2^)^1/2^, where 0.1 is the Ni–Fe–H signal rate, and 0.03 is the dark count rate. In order to let the real S/N reach 90% of the best S/N = 3.3, the following condition must be satisfied: 0.1t/((0.03t)^2^ + 0.1t)^1/2^ > 3.3 × 90% = 3 → t > 427 s per point (s/p). Our above sectional scan had 900 s in total in the Ni–H–Fe region.

Assuming 0.03 cts/s for the dark count level, we can set its doubled value (or 0.06 cts/s) as the realistic lowest cts/s limit for the NRVS signal. For the lowest sample or ^57^Fe concentration, it also depends on several other factors, such as the incident beam flux, APD area, and APD–sample distance. In addition, which peak we are talking about also varies substantially for the lowest detectable concentration. For example, according to our previous estimation, there would be 100 cts/s at the nuclear resonant peak measured at BL19LXU for a 1 mM ^57^Fe concentration. Even if 1 cts/s is set as the detection limit for measuring the resonant peak, the detectable limit would be as low as 0.01 mM ^57^Fe. However, measurement at this concentration does not lead to any vibrational peak. For measuring relatively strong vibrational peaks such as Fe–S/P/O, there is the following general understanding about the lowest detectable ^57^Fe at SPring-8: 1 mM at BL09XU or 0.3 mM at BL19LXU. On the other hand, the corresponding sample in Figure 1b is a ^57^Fe-labeled 4.5 mM *DvM*F NiFe hydrogenase (11 irons in FeS clusters and 1 iron in the NiFe center), which leads to a total 4.5 × 12 ≈ 54 mM total ^57^Fe. We then consider 54 × (0.06/0.1) = 32 mM as the lowest total ^57^Fe concentration (or 2.7 mM as sample concentration) to measure the Ni–H–Fe wag mode at BL09LXU (for the particular hydrogenase enzyme). For measuring Fe–CO/CN in Figure 1b, experimenters need to provide samples with about 12 mM in total ^57^Fe concentration (or 1 mM in sample concentration). Of course, these are just figurative numbers for new users to have a general idea. The real detection limits depend on several issues.

Weak NRVS features are often more informative than the strong features. For example, in *DvM*F NiFe hydrogenase, the Fe–CO and Ni–H–Fe features are much more valuable than Fe–S features [7]. Practical options to further improve NRVS signals include (1) a higher ^57^Fe concentration inside the sample, (2) a stronger incident beam flux, (3) an asclose-as-possible sample–detector distance, and (4) a longer measurement time. Options (1) and (2) increase the sample–beam interaction, while options (3) and (4) allow collecting more signals.

### 3.4. Making and Monitoring Samples

Natural abundance Fe contains 2.14% ^57^Fe; thus, it is important that all samples are enriched with the probe isotope, e.g., ^57^Fe, for an NRVS measurement. Just to give the reader some idea on the involved cost of obtaining the necessary isotopes for NRVS sample preparation, 1 g of 99.9% enriched ^57^Fe may cost around 6700 CHF (6150 EUR/7250 USD) on the market. Alternatively, ^119^Sn may cost 7860 CHF (7200 EUR/8500 USD) per gram. The procedures for enriching alloys, as well as inorganic complexes with ^57^Fe (or other NRVS isotopes), will be as varied as the synthetic methods used. Enrichment of biological samples can also follow several routes, depending on the level of control over the site of interest. The most basic way is to enrich the growth medium for the organism that is producing the enzyme samples. For example, one can purchase or prepare soluble ^57^Fe-enriched salts, such as ^57^FeCl_3_ or ^57^FeSO_4_, and add them to an Fe-free growth medium. Alternatively, one can dissolve elemental ^57^Fe in *aqua regia* and add it to the growth medium. In the end, researchers can then extract the enzyme from the ^57^Fe-enriched organism. In addition to enrichment of the entire sample, one can also enrich just one selected iron site by maturation, which is a very delicate form of contrast enhancement. The selective labeling of a site of interest inside a large multi-cofactor-containing biological sample, such as [2Fe]_H_ in Figure 2a, will help reduce unwanted counts from other cofactors and make the regions of interest more obvious [47]. Of course, the site-selective labeling involves a better understanding and utilization of the detailed biosynthesis process [47].

On the other hand, while strongly discouraged by beamline scientists, in some cases, unenriched samples must be run with much longer time, like naturally isolated molecules or meteorites (for the rationale of the latter, compare the case for cosmic dust [74]).

**Figure 8.**
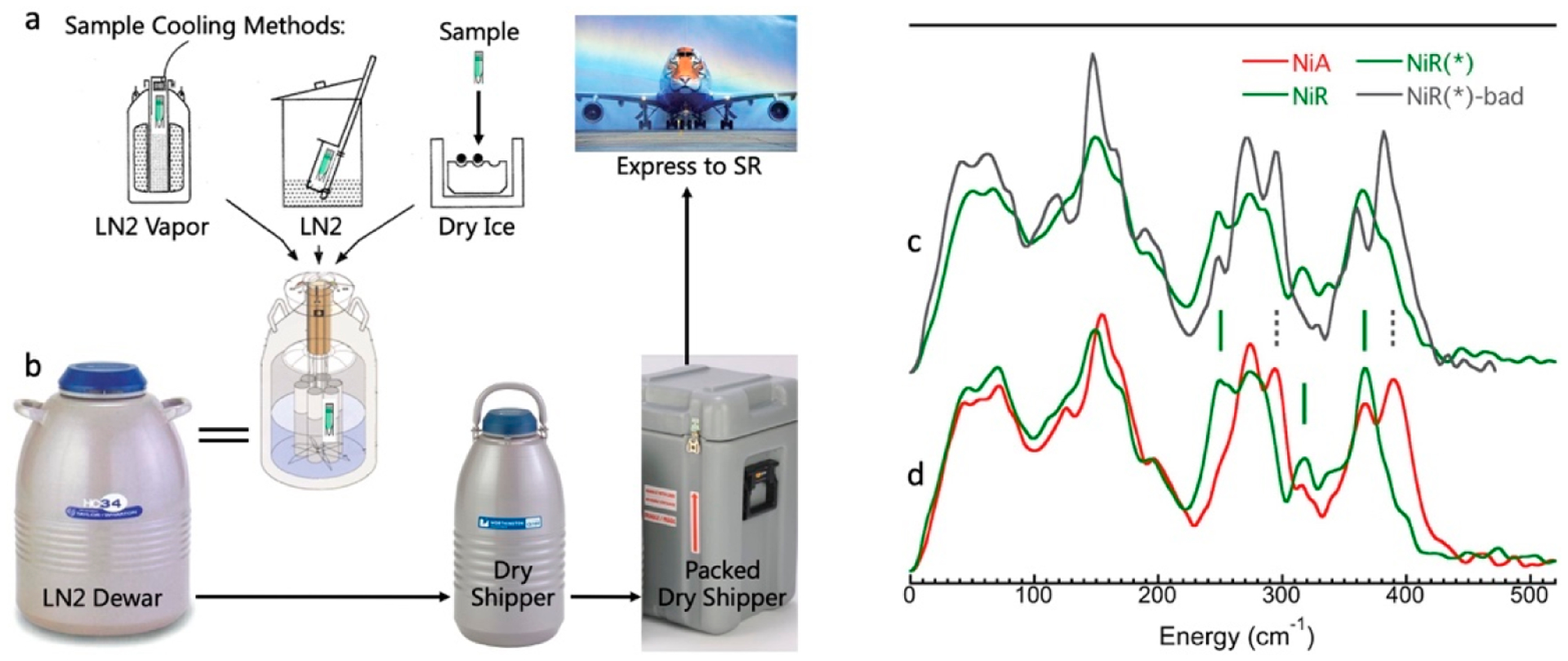
(**a**) Illustration of sample freezing → storage process; (**b**) illustration of sample shipment process; (**c**) the NRVS spectra from two different samples—NiR (*) is a good NiR sample while NiR(*)-bad is one which was accidentally oxidized; (**d**) the reference spectra for the oxidized (NiA) and reduced (NiR) *DvM*F hydrogenases.

Prepared solution samples were injected into a Lucite sample cell inside an oxygen free glovebox, frozen with liquid nitrogen (LN_2_) or LN_2_ vapor or dry ice, and then stored inside an LN_2_-filled sample dewar at 77 K, as illustrated in Figure 8a. Prior to the NRVS measurements, the samples were shipped using an express service to the SR center with a dry shipper at 77 K (but without LN_2_) (Figure 8b).

In addition to ^57^Fe (or any other relevant isotope) labeling, there are several sample conditions that make measurements on weak NRVS features feasible, whereby sample concentration is the most important. However, achieving high concentrations for big molecules can be complex. For example, a typical [NiFe] hydrogenase has a molecular weight of ~90 kDa; thus, 1 g of a 4 mM sample will consist of 360 mg of enzyme in 640 mg of H_2_O, leading to 36% (*w*/*w*) enzyme concentration in solution, which is nearly at the precipitation limit. On the other hand, this concentration only leads to 12 × 57 × 10^−3^ mg ^57^Fe/1000 mg total sample = 7 × 10^−4^ (*w*/*w*) in the total ^57^Fe concentration and 1 × 57 × 10^−3^ mg ^57^Fe/1000 mg total sample = 6 × 10^−5^ (*w*/*w*) for the ^57^Fe inside the NiFe center. Therefore, researchers must concentrate the sample as much as possible, on one hand, while they have to carefully control and monitor the sample’s integrity, on the other hand. A multiple spectroscopy adaptive sample holder (cell) is part of the requirement for biological NRVS measurements, where the same samples can be characterized with electron paramagnetic resonance (EPR), Mossbauer, or EXAFS prior to and following the NRVS measurement. For convenience, FTIR on the parallel solution prior to the sample shipments, as well as the redissolved sample after the NRVS experiments, can also provide straightforward information about the samples.

Preparation of inorganic samples can be less delicate. An example of a ceramic samples comes from the exploration of synchrotron-based ^61^Ni Mössbauer outlined in [75]. One example is the ^61^Ni Mössbauer in nickel chromate ^61^NiCr_2_O_4_. The sample material was kept in a quartz tube with 2 mm inner diameter and 3 mm outer diameter. For the inorganic sample with high ^61^Ni concentration, an X-ray optical path of 2 mm was sufficient (the sample thickness = the length of the quartz tube). This corresponds to an overall sample volume of ~7 mm^3^. In contrast, for rubredoxin with ^61^Ni, a sample thickness of 10 cm (quartz tube length) was required because the concentration of Ni, irrespective the isotope, is low in this protein. The quartz tubes were received inside a massive copper slab with 1 cm × 1 cm cross-section and 20 cm length. The slab had a cavity with 3.3 mm size in order to contain the quartz tubes with 3 mm diameter.

Let us consider, for example, the proton-conducting ceramic BaSnO_3_, with ^119^Sn being the Mössbauer or NRVS isotope. With a density of around 7.3 g/cm^3^, an amount of around 50 mg fits in the 2 mm long quartz tube. According to the stoichiometry of Ba^119^SnO^3^, we need around 20 mg of ^119^Sn. The cost for the ^119^Sn isotope is around 140 EUR for such a small sample.

At the beamline, in situ NRVS monitoring can also be performed by observing scanto-scan changes in some low-energy strong signal features, such as the Fe–S bands. In Figure 8d, the green spectrum represents the NRVS for the reduced (NiR) state hydrogenase, while the red spectrum represents that for the oxidized (NiA) state hydrogenase [7,8]. In Figure 8c, the blue spectrum matches the NiR NRVS (green in (d)), showing that the corresponding sample is in a good condition, while the gray spectrum matches the NiA spectrum (red in (d)), indicating that the specific sample has already been oxidized and should be discarded. Although IR or other spectroscopies are also used to monitor the sample prior to or following the NRVS measurement carefully, in situ NRVS monitoring is also critical before researchers spend lots of beamtime on the sample to measure weak features. It can at least tell whether the sample’s redox state is still in good condition or not.

### 3.5. Real Sample Temperatures

**Figure 9.**
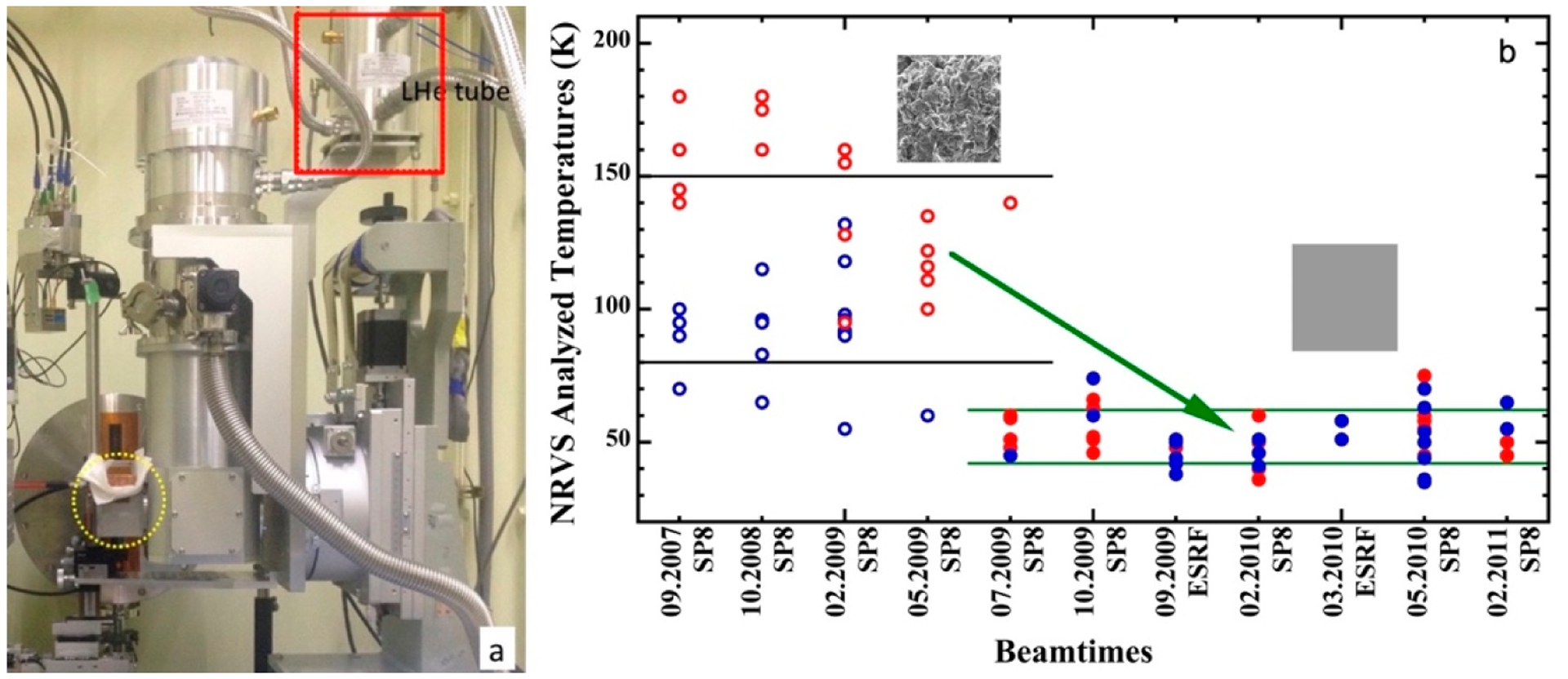
(**a**) Photograph of a closed-loop liquid-helium (LHe) NRVS cryostat at SPring-8 BL9XU with the sample space circled in yellow; (**b**) real sample temperature during different beamtimes from 2007–2011 at SPring-8 and ESRF [76]. Red is for biological samples and blue is for complex samples; filled circles represent samples that were attached to the cryostat by 1-propanol, and open circles are those that were attached by low-T grease.

As mentioned in Section 2.4, most NRVS samples are measured at an as-low-as-possible temperature, which is often maintained with a liquid helium (LHe) cryostat. To save the precious helium (He), closed-cycle He refrigerators are being installed in more and more SR beamlines. A photo for such a device used at SPring-8 BL09XU (and to be moved to BL35XU in the future) is shown in Figure 9a. With a two-stage He refrigerator system, enclosed He gas at RT is first compressed, and then supplied to the refrigerator via flexible gas lines. Then, the compressed He is cooled by expansion, providing cooling to the LHe supply station and continuing to the cryostat head at the sample location (dashed yellow circle). With this two-stage arrangement and the cushioned mechanical link between the He station and the cryostat head, the mechanical vibration is minimized for NRVS applications. However, it is still not good enough for nuclear forward scattering measurements where lesser mechanical vibration is required and a traditional He-flow cryostat will be used instead.

Although the cryostat base is cooled to an as-low-as-possible temperature and the sensor located near sample is often monitored at 10 K, the real sample temperature analyzed by the imbalance in the NRVS spectra is much higher.

There are two practices for mounting samples to the cryostat head: (1) to load the samples from LN_2_ onto the cryostat base at an as-low-as-possible temperature and fix the samples with screws (which is the practice at APS 03ID); (2) to use cryogenic adhesive to attach the sample onto the cryostat base (which is used at SPring-8 BL09XU and BL19LXU). The good aspect for (2) is that the time to mount the sample is very short (~15 s). However, the base temperature has to be maintained at a temperature above the cryogenic adhesive’s melting point. At the beginning, a cryogenic grease (also called low-T grease) was used as adhesive. At that time, different samples were loaded with different procedures: sensitive biological samples were loaded from LN_2_ onto a 180 K cryostat base (procedure A1), noncritical biological samples were loaded from LN_2_ onto a 190 K base (A2), and chemical complexes were most loaded from RT onto a 200 K base (A3). As illustrated in the left side of Figure 9b (not Figure 9a), (1) it has an overall temperature of 114 ± 34K, and (2) biological samples (red circles) show obviously higher temperatures than complex samples (blue circles). Although sample nature was first suspected, a series of tests later concluded that the real reason is that most biological samples (here) were loaded at 180 K when the low-T grease was almost in its solid state and had a point-to-point contact, as illustrated in the top left insert of Figure 9b [76]. To break down, the A1/A2/A3 procedure loaded samples have 154 ± 26 K/128 ± 20 K/63 ± 6 K, respectively. Later, organic solvent 1-propanol with a melting point at 147 K was selected as the new cryogenic adhesive, and the cryostat base was maintained at 150 K for sample loading. The contact becomes a surface-to-surface contact as shown in the lower right insert of Figure 9b [76], leading to a much lower average temperature at 52 ± 10 K.

X-rays can cause radiation damage to sensitive samples [77,78] by heat dissipation. The rate of the radiation damage reaction is, thus, proportional to exp[−(1/kT)]. According to Garman et al. [78], large movements of atoms would be suppressed at 100 K because the amorphous solvent at cryo-temperatures is a glass with rigidly bound atoms. Even for locally sensitive EXAFS, much less radiation damage was found at temperatures of 7–40 K in comparison with that at 100 K [78] (but not much difference between 7 and 40 K). Therefore, lowering the real sample temperature from 154 ± 26 K/128 ± 20 K to 52 ± 10 K is a significant step toward controlling the possible radiation damage, which will be great preparation for future NRVS measurements with higher photon flux. For the NRVS measurements since 2012, we have an overall temperature ranging from 30 to 60K for almost all the NRVS samples.

### 3.6. Photolysis and NRVS

For some samples, photolysis experiments are required to produce the sample in the needed state. Photolysis NRVS experiments can be performed with an in situ setup where the photolysis light is shone in from the slim space between the sample’s surface and the APD detector via a wave guide to the transparent side window, as mentioned in a previous publication [46]. An alternative photolysis NRVS measurement procedure uses a transparent window in front of the sample’s surface and does photolysis at a cryostat temperature without NRVS measurement. Afterward, the photolysis setup is removed and the APD detectors are put on for NRVS measurement. Each option has its own drawbacks. The former option has a longer sample-detector distance and/or lower photolysis efficiency. The latter could have part of the photolyzed sample returned to its pre-photolysis state before or during the NRVS measurement.

## 4. Experimental Aspects (III): Data Process

### 4.1. Converting to PVDOS

As with the analysis of other spectroscopic data, researchers need first to perform preliminary data processing on the experimental data, which includes (1) normalizing the spectrum by the incident X-ray intensity I_0_, and then rescaling the spectrum to its original counting level, (2) calibrating the nuclear resonant peak position to the energy’s zero-point E_0_ = 0, and (3) summing the overall counts of multiple spectral scans. Such an obtained “final” spectrum is the relationship between the spectral counts and the vibrational energy positions, which is a summed raw NRVS spectrum. For a better theoretical understanding (to obtain the force constants and structural information); researchers also need to convert these raw NRVS spectra into the ^57^Fe PVDOS (or *g*(*w*) in physics or spectroscopy textbooks). Unlike other vibrational spectroscopies, NRVS is able to obtain a real PVDOS. The most popular software packages available for processing raw NRVS data → PVDOS are PHOENIX [17] and DOS [79]. In addition, NRVS data from SPring-8 need to be first converted into a PHOENIX-compatible format before processing with the software. The very basic conversion include re-sorting the data from high→low energy direction to low→high direction and putting energy in nuclear resonant transition scale with keV instead of vibrational scale with meV. Gee [14,80] combined the format conversion and PHOENIX analysis in a web program at http://spectra.tools, (accessed on 1 August 2021) to load and process the SPring-8 data directly.

**Figure 10.**
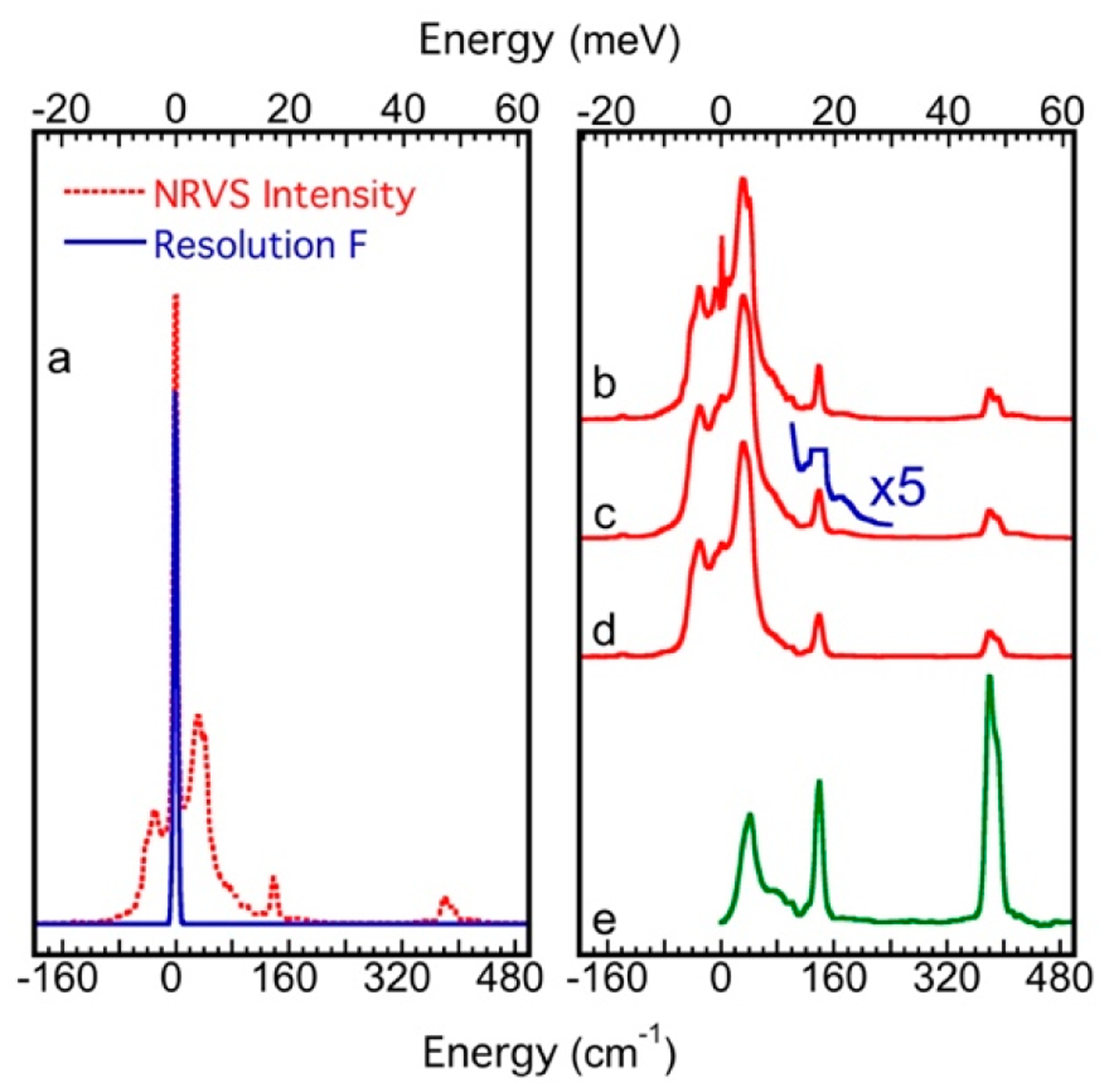
The analysis flow chart of raw NRVS → PVDOS (**a**–**e**) using [NEt_4_][FeCl_4_] as an example. Please refer to the text description in this section for details of the analysis process (**a**–**e**). The horizontal axis is the vibration energy; the lower axis unit is cm^−1^, which is commonly used in chemistry, while the upper horizontal axis unit is meV which is often used in physics or materials sciences.

The conversion from raw NRVS data to PVDOS includes the following major steps [17,46,79], as illustrated in Figure 10a–e:

1. The summed raw NRVS (*N(E)*) is actually the convolution of a theoretical energy spectrum *S*(*E*) (with zero bandwidth) and the energy linewidth profile, also called the resolution function *R*(*E*).
(5) N(E)=S(E)×R(E).
  The resolution function *R*(*E*) can be measured with nuclear forward scattering on a piece of α–^57^Fe foil at the NRS beamline or by selecting a generic Gaussian distribution in the analysis software. The first step of the analysis is to deconvolute *N*(*E*) into *S*(*E*).
2. At this point, the nuclear resonant peak still exists but has become “a line”, as shown in Figure 10a–b and summarized in Equation (6) (in reference to Equation (1)).
(6) $$S(E)={\text{f}}_{\text{LM}}\left\{\delta (E)+\Sigma S_{\text{n}}(E)\right\}.$$
  The nuclear resonant peak *δ(E)* is removed, while considering the effect of the LM factor f_LM_ to obtain a pure inelastic scattering spectrum Σ*S*_n_(*E*) (see the steps of b–c). The Σ*S*_n_(*E*) is the pure contribution from the vibrational scattering, but it contains the contribution from both single-phonon and multi-phonon processes.
3. The contribution from multi-phonon processes can be determined and removed, while extracting the contribution from the pure single-phonon scattering spectrum *S*_1_(*E*) (d). It is obvious that multi-phonon effects exist in the NRVS spectra, e.g., the artefact peak at 21.5 meV shown at ×5 in Figure 10c. This might be accidentally considered as a vibrational mode if the multi-phonon effect is not removed properly.
4. The single-phonon spectrum is in the form of Equation (7).
(7) $$S_{1}(s,E)=E_{\text{R}}\times g(s,\left|E\right|)/\left\{E\left(1-{\text{e}}^{-\beta E}\right)\right\}$$
  where *S*_1_(***s***, |*E*|) is the function of direction ***s*** and energy *E*. If the sample is isotropic, Equation (7) becomes Equation (8).
(8) $$S_{1}\left(E\right)=E_{\text{R}}\times g(s,\left|E\right|)/\left\{E\left(1-{\text{e}}^{-\beta E}\right)\right\}$$

From Equation (8), people can consider the influence of sample temperature and convert the Stoke and anti-Stoke spectral branches into the final PVDOS (steps (d) → (e)). Of course, this includes all normal vibrational modes with ^57^Fe in motion.

Once the NRVS-derived PVDOS is in hand, the interpretation depends on the problem of interest. Due to the simplicity of the selection rule, many features in an NRVS spectrum can be initially assigned on the basis of a combination of intuition, literature precedents, and symmetry analysis [5,29,30,46,81]. To further refine the assignments experimentally, ligand isotopic substitution (e.g., ^12^C → ^13^C [9] or ^34^S → ^36^S [37]) can be used to highlight the particular mode(s) involved. Different redox states for the same sample are also an important source of spectral comparison. A set of initial force constants can be optimized to “the real force constants” when the calculated PVDOS reproduces the observed one in a normal mode analysis (NMA) [50,81]. In addition, NMA also allows calculating thermodynamic parameters, e.g., vibrational entropy and mean vibrational energy. Alternatively, density function theory can provide an ab initio calculation. In short, the following are some steps that people can carry out with the NRVS-derived PVDOS:

- sum rule analysis for LM factor, etc.,
- chemical shifts and isotope shifts,
- interpretation via empirical NMA simulation,
- interpretation via ab initio DFT calculations.

### 4.2. Understanding Vibrational Modes

**Figure 11.**
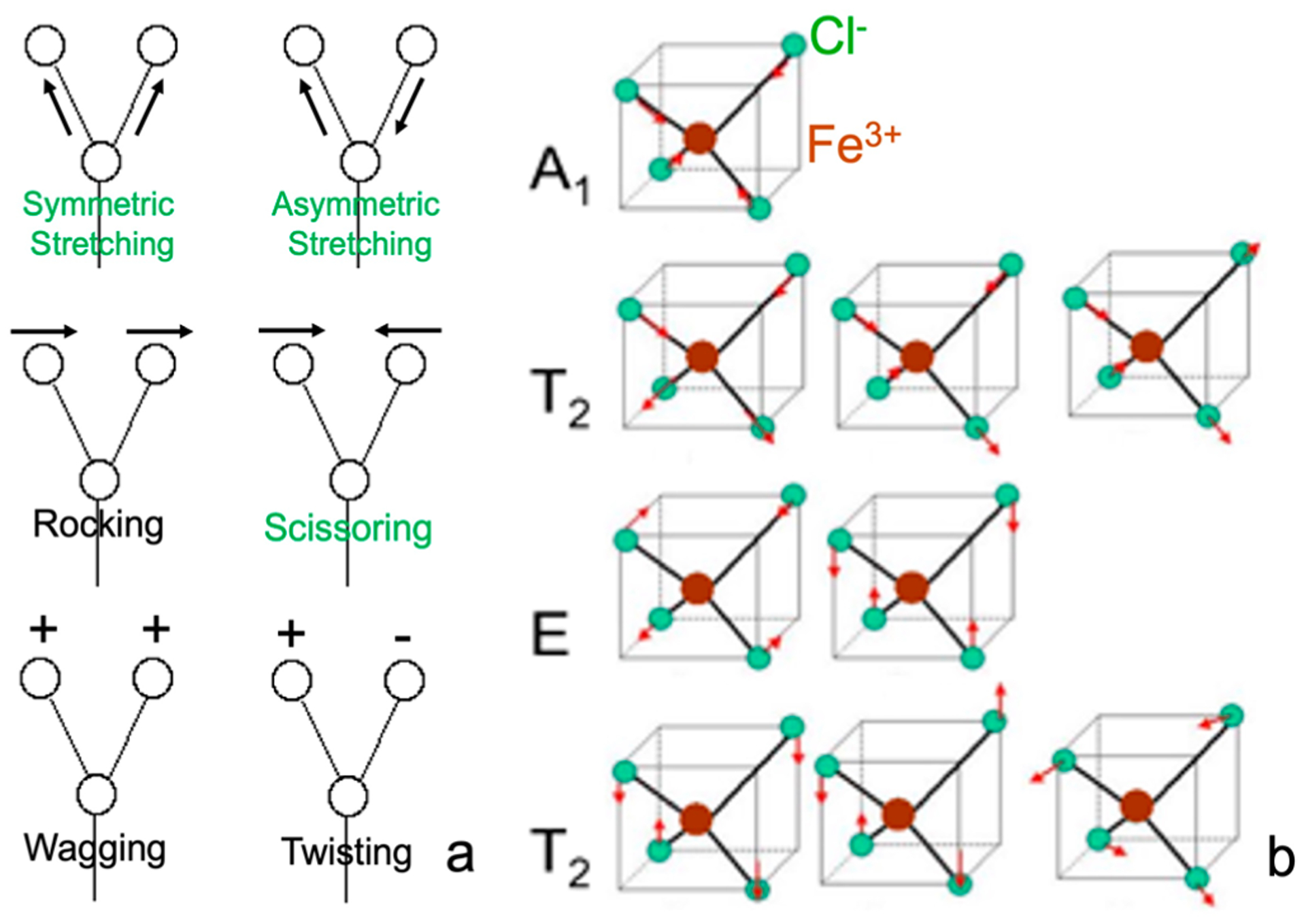
(**a**) Localized three-atom vibrational modes inside a big molecule; (**b**) nine normal vibrational normal modes in five-atom [FeCl_4_]^−^ ion and their sub-symmetries.

The ultimate goal of NRVS is to measure the vibrational modes; hence, it is necessary to understand the basics about molecular vibrations. A diatomic molecule contains a single vibration—stretching mode, while polyatomic molecules exhibit more complex vibrations. If a three-atom structure is localized inside a big molecule, there are six vibrational modes available: asymmetric stretching, symmetric stretching, wagging, twisting, scissoring, and rocking, as illustrated in Figure 11a. However, these localized vibrational modes are not synchronized with the vibrations in other parts of the big molecule. Vibrations in different parts of the molecule will have different frequencies and different positional phases. Therefore, localized vibrations cannot be studied with vibrational spectroscopy. People have to create a new set of vibrational coordinates, which are the linear combination of all localized vibrational coordinates in the whole system (*e.g.,* one molecule) and rerepresent the localized vibrations as the whole molecule’s vibrations. Such “transformed” vibrations are independent of each other and called normal mode vibrations. While different modes have different vibrational frequencies, each mode has one vibrational frequency and the same motion phase for all atoms, meaning that all atoms pass their balance position at the same time and reach their maximum displacement point at the same time.

As each normal vibrational mode has one vibrational frequency, it corresponds to one peak in the measured vibrational spectrum if the transition is not forbidden. Due to the symmetry in the molecular structure, as well as that in its electric field, multiple normal modes may share the same energy level and, thus, share one peak. This is called degeneracy. For example, the [FeCl_4_]^−^ ion has five atoms and nine normal modes (3N − 6 = 3 × 5 − 6 = 9), which can be classified into the following four categories due to ion its tetrahedral (T_d_) symmetry, as shown in Figure 11b: (1) the symmetric stretching mode, with A_1_ symmetry, which is non-degenerated, meaning that there is only one vibrational mode at this vibrational energy; (2) the asymmetric stretching mode with T_2_ symmetry and a threefold degeneracy, meaning that there are three vibrational modes at the same vibrational energy; (3) the symmetric bending mode with E symmetry and a twofold degeneracy, meaning that there are two vibrational modes at this energy; (4) the asymmetric bending mode with T_2_ symmetry and a threefold degeneracy, meaning that there are three vibrational modes at this energy. In the above vibrational modes, symmetric modes mean that the Cl^−^ is moving in or out symmetrically, leaving ^57^Fe in the center not moving and, therefore, having no NRVS signal. On the other hand, asymmetric modes mean that some Cl^−^ ions are moving in while others are moving out; ^57^Fe is also in motion and, therefore, has an NRVS signal. After getting the vibrational spectrum for normal modes, people can then reconnect them to the localized chemical bonds, such as Fe–Cl, Fe–S, and Fe–C, via spectral simulation.

The above analysis assumes that molecules are independent of each other (in a gas phase-like state). In solids, the molecules (or unit cells) in the lattice are all interconnected with each other. For example, an NaCl crystal has Na^+^ and Cl^−^ in the lattice cell. However, the vibration is certainly not limited to one Na–Cl pair. The vibrations involve all other atoms inside the whole crystal lattice. Then, the lattice vibrations can be divided into optical modes (e.g., between Na–Cl) and acoustic modes (between different NaCl cells in long range). The above-discussed nine normal modes for the [FeCl_4_]^−^ ion represent the vibrations inside one molecular cell and belong to optical modes, which have relatively higher and scattered vibrational energy levels. In addition, there are peaks which describe the whole lattice’s acoustic modes, vibrations due to interactions between the [FeCl_4_]^−^ and the counter ion or between the different [FeCl_4_]^−^ ions, or vibrations due to nonbinding weak interactions (such as hydrogen bonds). These modes are all in the near-zero energy region, such as the continuous features <100 cm^−1^ in Figures 1b, 2b, 8c,d, and 10e.

In general, vibrational normal modes for complicated molecules can always be categorized into symmetric stretching, asymmetric stretching, symmetric bending, asymmetric bending modes, acoustic modes, and nonbonding interaction modes.

### 4.3. Normal Mode Analysis (NMA)

Under the condition of small vibrations (harmonic vibrations) and treating molecules as a mechanical model, the vibration energy (frequency) can be written as

(9) $$V=\sum_{i}\frac{1}{2}{\text{K}}_{\text{i}}{\left(\Delta r_{i}\right)}^{2}+\sum_{i}\frac{1}{2}{\text{H}}_{\text{i}}r_{i\alpha }{}_{\text{i}}^{2}\left(\Delta \alpha_{i}\right)+\sum_{i}\left\{\frac{1}{2}{\text{F}}_{\text{i}}{\left(\Delta q_{i}\right)}^{2}+\frac{1}{2}{{\text{F}}^{\prime }}_{\text{i}}q_{\text{i}}{}^{2}\left(\Delta q_{i}\right)\right\}.$$

The first item represents the stretching vibrations, where Δ*r*_i_ represents the elongation or compression of chemical bonds; the second represents the bending vibrations, where the Δ*α*_i_ represents bond angle deformation; the third represents the long-distance or nonbonding vibrations; *q*_i_ and Δ*q*_i_ are the vibrational distances and angles in a broad sense—often used to describe nonbonding interactions. One of the most prominent examples of nonbonding interactions is the hydrogen bond. The frequencies of all normal mode vibrations discussed in the last section can be well calculated using Equation (9); thus, this is called normal mode analysis (NMA). By matching the calculated frequencies with the measured ones, NMA can obtain a series of force constants and, therefore, the structural information for the corresponding molecule, which is true for all vibrational spectroscopies.

In addition to the frequency simulation described by Equation (9), NRVS (PVDOS) intensity can be calculated using Equation (2). The frequency–intensity simulated force constants from NRVS should be more reliable than those obtained with the frequency-alone simulation from IR and RR spectra. Therefore, this becomes one of the prominent advantages of NRVS. NMA is an empirical simulation. Its input parameters include a series of trial (empirical) force constants in addition to the crystal structure, which can be the whole molecule structure, the optimized/more symmetric structure, or even a simplified central structure around the ^57^Fe. People often start with a simple structure and add more ligand layers step by step to gradually improve the simulation and obtain reliable force constants. The calculations using molecules with poor symmetry often need to include up to 4–5 layers of ligand [5] or even the entire molecule [82]. Sometimes, it is necessary to include the interaction between multiple layers of atoms at one time, not just adding them layer by layer; sometimes, it is necessary to treat the same type of atoms at different geometric locations as different “atoms” and deal with them separately. In short, the details of the calculation process vary from one chemical system to another.

NMA simulations are inherently local. As the simulated system increases in size, the numbers of bond lengths, angles, force constants, etc. become untenable to fit and the variables tend to become correlated. In addition to the empirical NMA simulation, ab initio quantum chemistry calculations can link NRVS spectra to molecular structure(s).

### 4.4. Density Function Theory (DFT)

The earliest idea for quantum chemistry calculation originated in the 1920s, see for example the works by Heitler and London, and by Dirac [83,84]. However, traditional quantum methods require relatively large calculations to deal with the interaction between the 3N coordination variables in an N-electron system. The primary contribution of density function theory (DFT) is to simplify the N electrons in an electron sea shared by all atoms and without electron–electron interactions, therefore, reducing the calculation load dramatically. The establishment of the two Hohenberg–Kohn theorems (H–K theorems) in the 1960s laid the foundation for today’s DFT. The first H–K theorem indicates that a multi-electronic system can be determined solely by the distribution of electronic density n(***r***), i.e., a three-dimensional function, while the second one defines the energy function of the system and proves that the correct n(***r***) minimizes the energy for the whole system.

To understand the basic principle (for calculating a molecule), one can start with a Schrodinger equation, in which the Hamiltonian does not include the Hartree interaction U_H_ and exchange interaction U_XC_ (the interactions between electrons) and obtain the wave function *ϕ*_i_(***r***) based on that. Then, the electronic density distribution function n(***r***) can be calculated from the wave function *ϕ*_i_(***r***). Effective U_H_, U_XC_ items can be constructed using n(***r***) and added to the potential energy *V*_s_(***r***). Next, we can bring the new *V*_s_(***r***) back to the Schrödinger equation and get to the next round of calculation, Schrodinger equation → *ϕ*_i_(***r***) → *n*(***r***) → (*U*_H_+*U*_XC_) → *V*_s_(***r***) → Schrodinger equation → *ϕ*_i_(***r***) → *n*(***r***) → … This iterative method can be continued until the process reaches a predetermined degree of convergence.

A DFT calculation requires the structural coordinates, total charge/total spin for each atom, and outputs the energy distribution for the whole system. For NRVS, first principles calculations of PVDOS are derived from the orthogonal matrix that diagonalizes the mass-weighted second-derivative Hessian matrix [85]. The procedure is regularly implemented in many quantum chemistry programs for calculating the vibrational frequencies and intensities for conventional spectroscopies (e.g., infrared, Raman). For NRVS specifically, PVDOS is calculated from the vibrational displacements, which captures the normal mode composition factors of the probe nucleus determined by DFT for each normal mode. By comparison to the experimental PVDOS, the displacements and frequencies of the calculated vibrational modes then afford deep insight into the nature of specific experimental features. Furthermore, DFT can reveal vibrational coupling between the probe atom(s) and nonlocal/diffuse vibrations.

DFT is a relatively specialized calculation, which is often carried out via collaborative research with theoreticians specialized in this field. The primary principles that experimenters need to recognize are as follows: (1) in comparison with NMA, DFT uses no empirical parameters for the system of study and can, thus, obtain a more reliable model structure along with more reliable force constants; (2) due to challenges in accounting for anharmonicity, DFT calculations often require the energy axis (normal mode frequencies) to be rescaled in order to obtain a reasonable match with the observed NRVS. A rough recommendation is that NMA should be utilized for smaller/high-symmetry systems where the size of the parameter space can be reasonable and group theory insights are useful. However, in recent years, DFT calculations have been popular as a straightforward approach to interpret vibrational spectra including NRVS [7,14,30,32,36,38,44,66,68].

## 5. NRVS Applications

NRVS is exciting, in part, because it can pinpoint the ^57^Fe-related vibrational modes without a “messy” background. Below, we select a few examples to illustrate NRVS applications (or possible applications) in various areas.

### 5.1. Examples in Materials Sciences

One application of nuclear resonant vibration spectroscopy is the determination of the phonon density of states in condensed matters. A class of materials of interest is the strongly correlated fermion systems, for example, those which show colossal magnetoresistance. Some of these are also interesting for electrochemical energy conversion in high-temperature ceramic fuel cells (solid oxide fuel cells, SOFC) and gas diffusion electrodes in batteries. When Fe is a constituent in such compounds, NRVS becomes applicable. An early example of NRVS was presented by Rykov et al. who studied the effects of magnetic order and electron transport coherence on the phonon density of two iron perovskites (Sr_2_FeCoO_6−δ_ and BaSrFeCoO_6−δ_) [86]. The samples were present in powder form, obtained by sol–gel synthesis involving a 20% ^57^Fe-enriched Fe_2_O_3_ [87].

For a relevant experiment, to establish the measuring nuclear resonances of nickel, Gee et al. applied synchrotron Mössbauer spectroscopy and studied ^61^NiCr_2_O_4_, which has a spinel structure [75]. The material was produced by a ceramic synthesis method—by mixing powders from Cr_2_O_3_ and a not further specified compound containing a 99% enriched ^61^Ni isotope in an agate mortar, with subsequent heating in an air vented at 1200 °C. This is a synthesis method typically applied in lithium battery research and other fields as well, where ceramic components are functional. With ^61^Ni nuclear forwarding spectroscopy or NRVS in the future, battery materials such as LiNiO_2_ and LiNiCoO_2_ can be evaluated. Meanwhile, systems which contain iron, such as a layered metal oxide LiNi_0.1_Fe_0.90_O_2−δ_ (LNF) [88] or iron-based superconductors [89], can be studied with the current ^57^Fe NRVS.

We are grateful to the anonymous reviewer who posed the question of whether it is possible to derive the Debye temperature from the PVDOS obtained with NRVS. For conventional Mössbauer spectra, this has been exercised by Sawatzky et al. [85], who noted, however, that the Debye temperature obtained from integration of Mössbauer spectra may be different from that obtained by calorimetry, possibly due to inadequacy of the Debye model for compounds with spinel and garnet structure [85]. It should nevertheless be possible to apply the same routines as exercised in [90,91] with synchrotron Mössbauer spectra. Although the NRVS spectra are element-specific and, thus, constitute only the isotope (for example, ^57^Fe) projected VDOS, Hu et al. have shown that the total PDOS can be extracted from the long wavelength properties [92]. Lin et al. used the relationship between Debye temperature and Lamb–Mössbauer temperature for the discussion of structural and electronic transitions that occur by variation of pressure in Fe_2_O_3_ [93].

### 5.2. Simple Chemical Complexes

**Figure 12.**
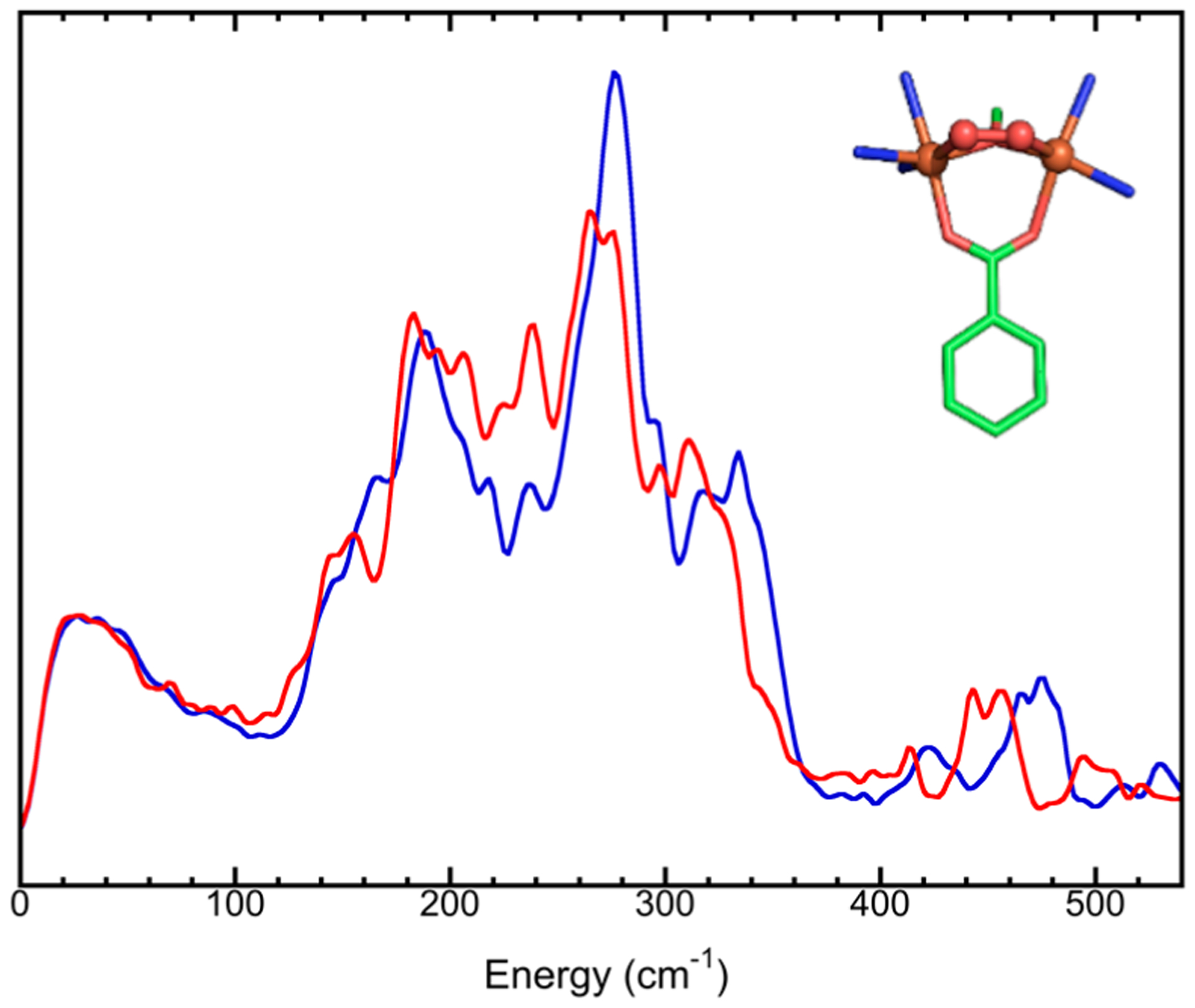
NRVS spectra for the ^57^Fe_2_(μ-O_2_)(N-EtHPTB)(PhCO_2_) (blue) and ^18^O-substituted compound (red). Inset, truncated image of the Fe_2_O_2_ site and neighboring atoms (just the bridging part).

To provide one possible model for biological peroxo bridges, we first introduce a peroxo diiron complex, [Fe_2_(μ-O_2_)(N-EtHPTB)(PhCO_2_)]^2+^ (abbreviated as Fe_2_O_2_, Figure 12 insert) [94]. Its NRVS spectrum (Figure 12, blue) is dominated by the envelope of peaks at 150–300 cm^−1^ which are related to Fe-(N-EtHPTB/PhCO_2_). The features at 446 and 458 cm^−1^ in the natural abundance spectrum shifted to 446 and 458 cm^−1^ in the ^18^O sample (red). Likewise, the shoulder at 338 cm^−1^ also shifted to a lower energy position of 311 cm^−1^ in the ^18^O isotopologue, showing that these features are related to Fe–O vibrations in the bridge. With isotope shifts and empirical NMA analysis, the pair of highest-energy peaks were characterized as a Fermi doublet [42,95] dominated by Fe–O–O–Fe symmetric stretching motion. Again, NRVS does have peaks for symmetric vibrations when the irons are not in the center of the structure. The feature at 338 (311) cm^−1^ was understood via NMA simulation [81,94] as a coupled O–O motion in parallel to the Fe–Fe axis and perpendicular to a pseudo mirror plane bisecting the four Fe–O–O–Fe atoms, later referred to as a “peroxide rock”. The initial study did not elaborate on the modes in the 150–300 cm^−1^ region [94], but a later DFT calculation [96] described the region as a combination of “core motions”, a peroxide twisting motion, and a “butterfly” motion where oxygen ions move perpendicular to the Fe–O–O–Fe plane and in opposite direction to the motion of the iron ions. These studies established the basics for further investigation of biological binuclear iron oxygenases [97], especially the intermediates.

There are many other chemical complexes used to model biological sites or for other purposes. [NEt_4_][FeCl_4_] is among one of the simplest chemical complexes; its [FeCl_4_]^−^ ion has five atoms nine 9 vibrational modes (optical modes), as shown in Figure 11b. Its NRVS (Figure 10e) has an asymmetric Fe–Cl stretch at 380 cm^−1^, an asymmetric Cl–Fe–Cl bend at 140 cm^−1^, and one broad profile at <80 cm^−1^, which is a collection of weak or long-range interactions. Since the iron is in the center of the [FeCl_4_]^−^ ion, the symmetric vibrations do not have an NRVS signal. In Figure 2b, the [Fe_2_S_2_Cl_4_]^=^ ion has many NRVS peaks, including those corresponding to symmetric vibrations as its irons are not in the ion’s symmetric center; thus, the symmetric vibration can also involve iron motion. The peaks can in general be classified as stretching modes, bending modes, and long-range interaction modes at <80 cm^−1^. In the [Fe_2_S_2_Cl_4_]^=^ NRVS (Figure 2b), more than one stretching peak and more than one bending peak exist in comparison with [NEt_4_][FeCl_4_] (Figure 10e) because (1) the eight-atom ion has more (18) vibrational modes, (2) 16 out of 18 modes are NRVS-active, and (3) lower symmetry leads to less energy degeneracy. There are many more Fe–S complexes (from 1Fe to 8Fe) to provide models for iron–sulfur proteins.

### 5.3. A Simple Iron Protein: Solution and Crystal

**Figure 13.**
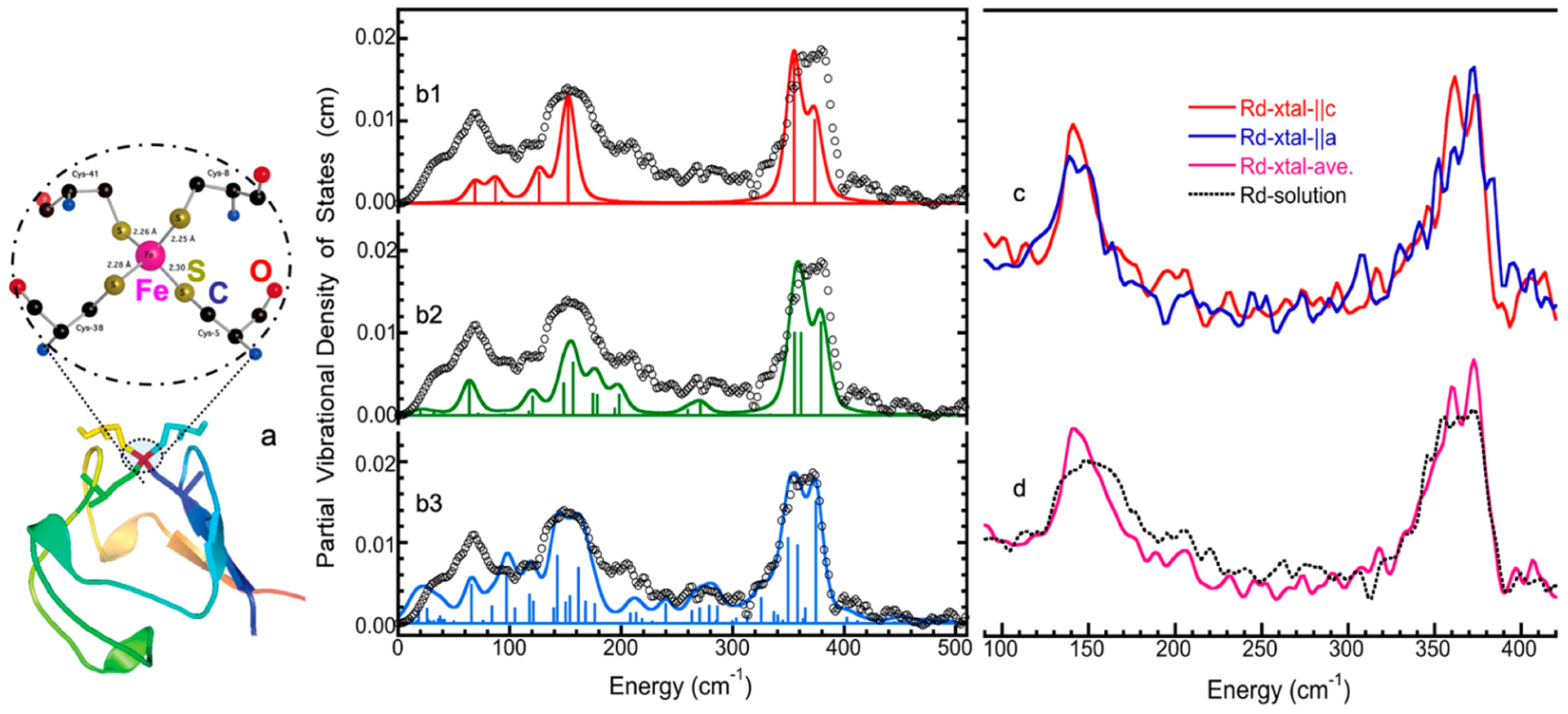
(**a**) The crystal structure of rubredoxin (bottom, PDB: 1CAD) and the Fe site inside rubredoxin and proximal cysteine chains (top, main atoms only); (**b**) the observed NRVS spectra of the oxidized rubredoxin protein (open circles) vs. the NMA simulations assuming an Fe(SC)_4_ structure (b1); an Fe(SCC)_4_ structure (b2) and an Fe(SX5)_4_ structure (b3) [5]; (**c**) a single-crystal NRVS with incident X-ray parallel to **a** (blue) and to **c** (red); (**d**) the averaged single-crystal NRVS (purple) vs. the solution NRVS (dashed black).

Rubredoxin, which contains a single Fe center coordinated to four cysteine amino-acid residues (SCCC …, S = sulfur, C = carbon), is considered one of the simplest metallo-proteins (6 kDa in molecular weight). The immediate coordination structure around the Fe includes four S, which is somewhat close to a T_d_ symmetry. The two accessible redox states of the Fe site are formally Fe(II) and Fe(III), which facilitate the electron transfer. The simplicity of the Fe site and the easy handling of this protein made rubredoxin an excellent testing candidate for many new bioinorganic spectroscopies, including ^57^Fe NRVS [5]. The crystal structure (a) and NRVS spectra (b1 → b3) for the oxidized protein are shown in Figure 13. Its observed NRVS (open circles in Figure 13b1 → 13b3) has peaks centered at 360, 150, and 70 cm^−1^, respectively, corresponding to stretching, bending, and long-range interactions. The reduced protein has peaks downshifted to 303, 140, and 70 cm^−1^ (not shown here [5]).

In comparison with Figure 10e, the three major rubredoxin NRVS profiles are a little bit similar to those for [FeCl_4_]^−^. However, significant differences exist. For the highly symmetric [FeCl_4_]^−^ ion, its normal modes are heavily degenerated, making the NRVS peaks clear and scattered. For rubredoxin, the NMA simulation assumes a single-layered Fe(S)_4_ structure in a T_d_ symmetry provides two symmetric peaks (not shown here), which is obviously far from the observed NRVS. Simulation with the Fe(SC)_4_ structure in a more realistic D_2d_ symmetry (b1) leads to six peaks to cover all three regions. However, the observed spectrum shows at least three lines in the main peak, many more lines in the 140 cm^−1^ peak, and obvious intensities in the region at 200–320 cm^−1^, while the simulation provides no transition. Further simulation with the Fe(SCC)_4_ structure in C_1_ symmetry (b2) provides a better simulation, and all the above problems are resolved. An Fe(SX5)_4_ structure with S and five additional layers of ligands [5] leads to a calculated spectrum which is almost the same as the observed NRVS (b3). This shows that the calculation on molecules with poor symmetry may need to include many layers [5] or even the entire molecule [82].

Via NMA, vibrational modes can be assigned. For the oxidized rubredoxin, the broad “peak” at 360 cm^−1^ is actually composed of three major peaks at 375, 358, and 350 cm^−1^ and three weaker contributions at 365, 364, and 340 cm^−1^, which are various Fe–S asymmetric stretching modes. The intensity in the shoulder from 300 to 340 cm^−1^ arises from the Raman-active and nearly symmetric Fe–S stretch modes [5]. A perfect symmetric stretch would not cause Fe motion; however, the contribution in this so-called “breathing” region includes about 10% from the asymmetric stretch components, which induces NRVS. The region at 150 cm^−1^ is composed of partially degenerate S–Fe–S bending modes. The region between 100 and 150 cm^−1^ is assigned as Fe–S–C bending modes but is also mixed with S–Fe–S bending modes. The <100 cm^−1^ region involves significant dihedral, torsional, acoustic, and delocalized vibrational modes within the longer-distance interactions.

In the original work [5], an empirical NMA was used to simulated both oxidized and reduced rubredoxin NRVS, although Figure 13 (b1) → (b3) only cites the work on the oxidized protein. The NMA simulations found a 36% decrease in Fe–S bond force constant (from 1.24 to 0.92 mdyne/Å) upon reduction of the protein, explaining the dramatic down shift of the Fe–S-related peaks around 360 cm^−1^ (→ 303 cm^−1^).

As discussed in the introduction, the vibrational modes of a single crystal of rubredoxin can be enhanced when the ^57^Fe motion is along the direction of the incident photon vector. As shown in Figure 13c, NRVS for single-crystal rubredoxin [98] has three strong asymmetric stretch modes at 373, 358, and 352 cm^−1^, corresponding to the modes at 375, 358, and 350 cm^−1^ in NRVS for the solution sample in Figure 13 (b1) → (b3). These peaks are selectively enhanced by orientation of the incident beam. The incident photon vector along the a-axis enhanced the peak at 373 cm^−1^, while the beam along the c-axis enhanced the 358 cm^−1^ peak (Figure 13c). Vibrational modes in the Fe–S bending region of the rubredoxin crystal were less orientation-dependent: the photon ∥c-axis enhanced the peak at 140 cm^−1^ and the photon ∥a-axis slightly split the peak.

Orientation-dependent NMA simulation with an Fe(SCC)_4_ C_1_ model was also performed to understand the single-crystal NRVS [98]. According to the simulation, (1) the vibrational mode at 352 cm^−1^ must lie perpendicular to the a-axis; (2) the enhancement of the vibrational mode with the highest energy (at 373 cm^−1^) is consistent with the beam ∥a-axis, which has shorter Fe–S bonds. The average of the two Fe–S bonds is 2.263Å along the a-axis and 2.284Å along the c-axis; (3) the structural difference between the two orientations is not great. This determined that the difference between their NRVS spectra is also small. Lehnert et al. published an article about [Fe(TPP)(Im)(NO)], where the complex showed a dramatic NRVS difference along different axes [99].

In addition to the orientational difference, the averaged single-crystal NRVS (solid purple curve) exhibits obviously sharper peaks in comparison with the solution NRVS (dashed black curve), as illustrated in Figure 13d.

### 5.4. FeS Cluster Compositions

Fe–S clusters have various forms, sizing from 1Fe to 8Fe. Please note, some do not consider 1Fe form as an iron-sulfur cluster. In iron-sulfur proteins, [2Fe2S], [3Fe4S], and [4Fe4S] clusters are the most frequent. Each cluster can have different redox states, and those in different organisms can also have a mild structural difference. For example, the [4Fe4S] cluster inside ferredoxin (e.g., *D*14*C*) and that inside HiPiP (high-potential iron–sulfur protein) have different structures and, thus, slightly different NRVS spectra. NRVS spectra for these FeS clusters were measured and published [9,29,36,37,67]. Using them as a database, the FeS clusters inside a new enzyme can be simulated. For example, *Ralstonia eutropha* soluble hydrogenase (or *Re* SH for short) had controversial “results” regarding the total number of irons inside this enzyme [29,100]. Sequence analyses indicated that the [NiFe] active center is wired to the NAD^+^-binding site by one [2Fe2S] and four [4Fe4S] clusters, as well as two flavin mononucleotide (FMN) molecules. The FMN cofactors were confirmed, but only one [2Fe2S] cluster and one [4Fe4S] cluster were detected by EPR [100]. In short, the number of irons or FeS clusters in *Re* SH is unclear. NRVS simulation can assist in this task.

**Figure 14.**
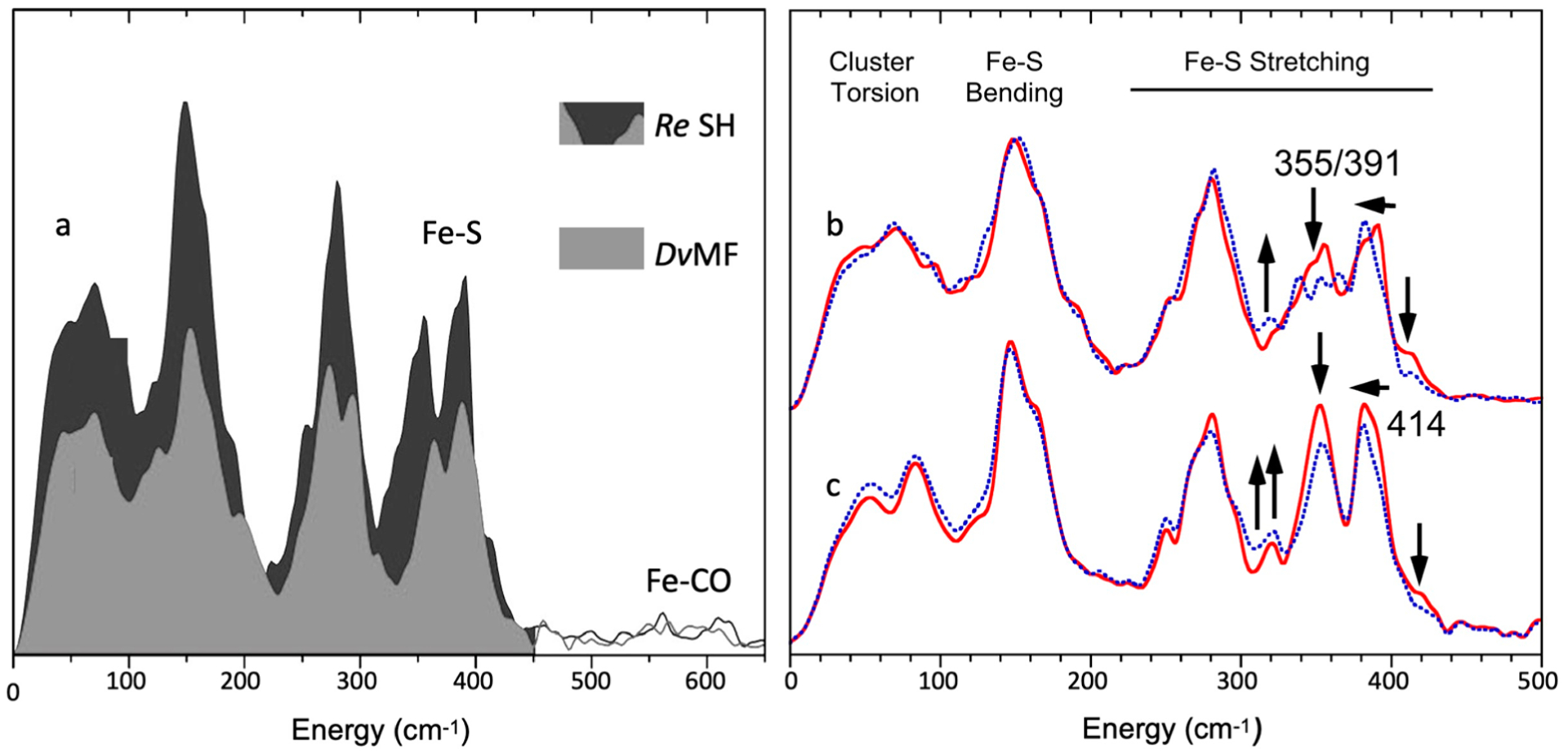
(**a**) Integrated areas for *DvM*F hydrogenase (dark gray shaded) and *Ralstonia eutropha* soluble hydrogenase (*Re* SH, black shaded) when their Fe–CO peaks are normalized to each other; (**b**) using four (oxidized) ferredoxin-like [4Fe4S] clusters and one [2Fe2S] cluster to simulate the NRVS for the oxidized *Re* SH; (**c**) using three oxidized and one reduced ferredoxin-like [4Fe4S] clusters and one [2Fe2S] cluster with partial reduction to simulate the NRVS for the reduced *Re* SH. The results are cited from [29].

First, the NRVS composition for *DvM*F NiFe hydrogenase which has one [NiFe] center (with one Fe–CO and two Fe–CN), one [3Fe4S], two [4Fe4S] clusters, and no [2Fe2S] clusters (11 irons in FeS) can be used as the known reference [9]. As both *DvM*F and *Re* SH have the same Fe–CO structure, we rescaled their NRVS spectra to let them have the same Fe–CO intensity. Then, we can assume that the integrated NRVS intensities in the FeS region are proportional to the iron numbers inside the FeS clusters in their enzymes [29]. The ratio of the two integrated intensities in Figure 14a (grey (*DvM*F) vs. black (*Re* SH)) is about 1.5, which is close to 18/11 ≈ 1.6, indicating that the sequence analysis could be correct while EPR results have a serious problem [29].

To further understand the issue, *Re* SH NRVS spectra were simulated with the combination of NRVS spectra from different FeS clusters. Figure 14b shows a nice compositional simulation on the oxidized *Re* SH with four oxidized *D*14*C* [4Fe4S] [36] and one oxidized [2Fe2S] cluster [37]. Other combinations were also tested in the original publication [29], but this was proven to be the best simulation. Figure 14c shows the best simulation on the reduced *Re* SH with three oxidized and one reduced [4Fe4S] [36], as well as with 0.5 oxidized and 0.5 reduced [2Fe2S] [37] (i.e., when the enzyme was reduced, only one [4Fe4S] and part of the [2Fe2S] were reduced, while all other FeS clusters were not reduced). As the oxidized [4Fe4S] and [2Fe2S] clusters have no EPR signal, EPR results for *Re* SH only show the reduced [4Fe4S] (1) and reduced [2Fe2S] (0.5), explaining the pre-NRVS controversy [29,100].

### 5.5. Fe–H Vibrations and Structures

**Figure 15.**
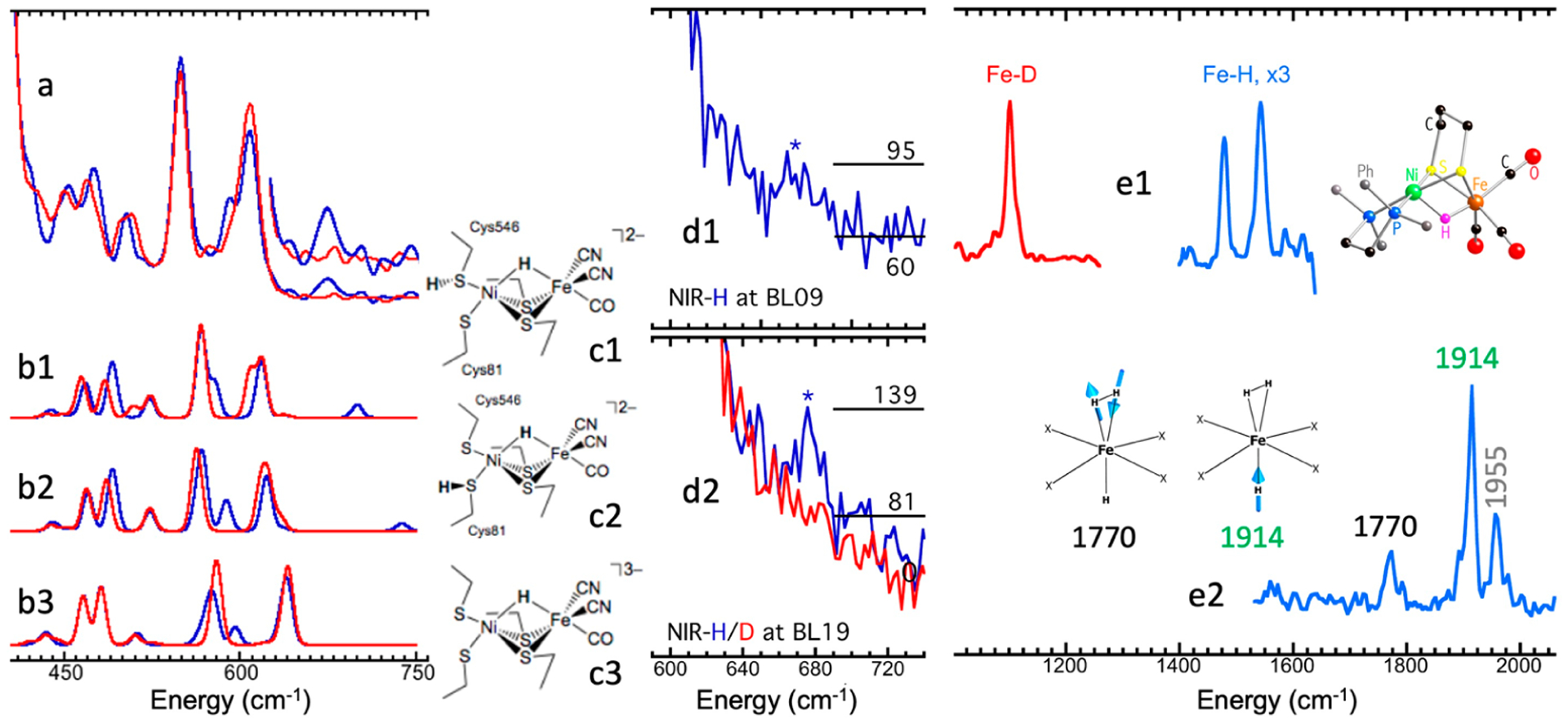
(**a**) NRVS for *DvM*F NiR–H/D (from Figure 1b); (**b1**–**b3**) DFT calculation on NiR–H/D assuming the structures in (**c1**–**c3**); (**d1**) the historical discovery of first Fe–H bending mode (raw NRVS) in a biological sample (*DvM*F hydrogenase); (**d2**) confirmation of the Fe–H bending (wagging) mode at higher flux BL19LXU; (**e1**) NRVS for the Fe–D/H stretching modes in an NiFe hydrogenase model complex (with model structure at the right); (**e2**) NRVS for various Fe–H stretching modes in HFe(H_2_) complex (with two vibrational modes at the left).

Figure 1b is the NRVS for *DvM*F. Among all the peaks, the 0.1 cts/s Ni–H–Fe wag mode at 627 cm^−1^ is the hardest to measure and most precious to have. Its existence and location provided a critical reference point for the structural identification of the NiFe center from many proposed DFT models [7]. According to Figure 15 (a vs. b1/b2/b3 and c1/c2/c3), the structure (c3) is clearly far from the real situation and should be ruled out. In cases where the information for the weak Ni–H–Fe peak is not available, the structural identification is almost impossible because all the three structures (c1, c2, and c3), as well as at least nine others in the original publication [7], have more or less similarly calculated Fe–CN/Fe–CO NRVS. The exclusive difference among them is the Ni–H–Fe peak which leads to the best match (c1 or c2). Nevertheless, all Fe–CN, Fe–CO, and Ni–H–Fe features are indispensable parts of the whole DFT calculations.

According to Section 3.3 and Figure 1b, Fe–CN/Fe–CO are still fine to observe with a signal level of about 0.7 cts/s. The Ni–H–Fe wag mode, on the other hand, is extremely hard to extract. The strenuous initial experiment to discover this peak at 627 cm^−1^ (Figure 15d1) [8] included two major aspects: (1) the sample concentration reached 4.5 mM, and (2) the experimenters made the right decision to search the lower energy region although all model complexes had Fe–H bending features around 750 cm^−1^ [7,101] while all DFT results at that time pointed to 800 cm^−1^ or higher. The further confirmation of this feature involved two more aspects: (3) a tripled incident X-ray intensity (with 0.8 meV energy resolution) at BL19LXU (vs. that at BL09XU), and (4) the H/D pair comparison, as shown in Figure 15d2. After the historical observation of this Ni–H–Fe peak, terminal X–Fe–H bending features inside several FeFe hydrogenses were also successfully observed, and their detailed structures were identified via DFT calculations [44,45,47,69].

In addition to the Fe–H bending features, Fe–D or Fe–H stretching features at high-energy regions stand out from all other features and can provide additional and better reference points for DFT calculation vs. proposed structures. Although it is still not possible to directly observe Fe–H/D stretching features in a dilute sample, the features inside various chemical complexes have been observed and published [7,68,102]. Figure 15e1 shows the best NiFe hydrogenase model complex so far, which has an Fe–D stretching peak at 1101 cm^−1^ and doublet Fe–H stretching features at 1476 and 1539 cm^−1^ [7]. The right insert shows the simplified crystal structure for this model. Figure 15e2 illustrated several Fe–H stretching modes and the NRVS for the trans-[^57^Fe(η^2^H_2_)(H)(dppe)_2_][BPh_4_] complex (HFe(H_2_) for short) [68]. The left inserts show the vibrational modes for the NRVS peaks at 1770 and 1914 cm^−1^; the former is the asymmetric Fe–H stretching mode from the Fe(H_2_) structure, while the latter is the Fe–H stretching from the FeH structure. The one at 1955 cm^−1^ is the Fe–H stretching mode from the residual molecule when the −H_2_ part is accidentally dissociated. Although it is a byproduct, this peak represents the NRVS peak with the highest energy so far.

### 5.6. Site-Selectivity Labeling

NRVS spectra are isotope-specific and thus site specific in general. However, here, we illustrate examples of being able to distinguish the features belonging to different ^57^Fe sites inside one molecule.

**Figure 16.**
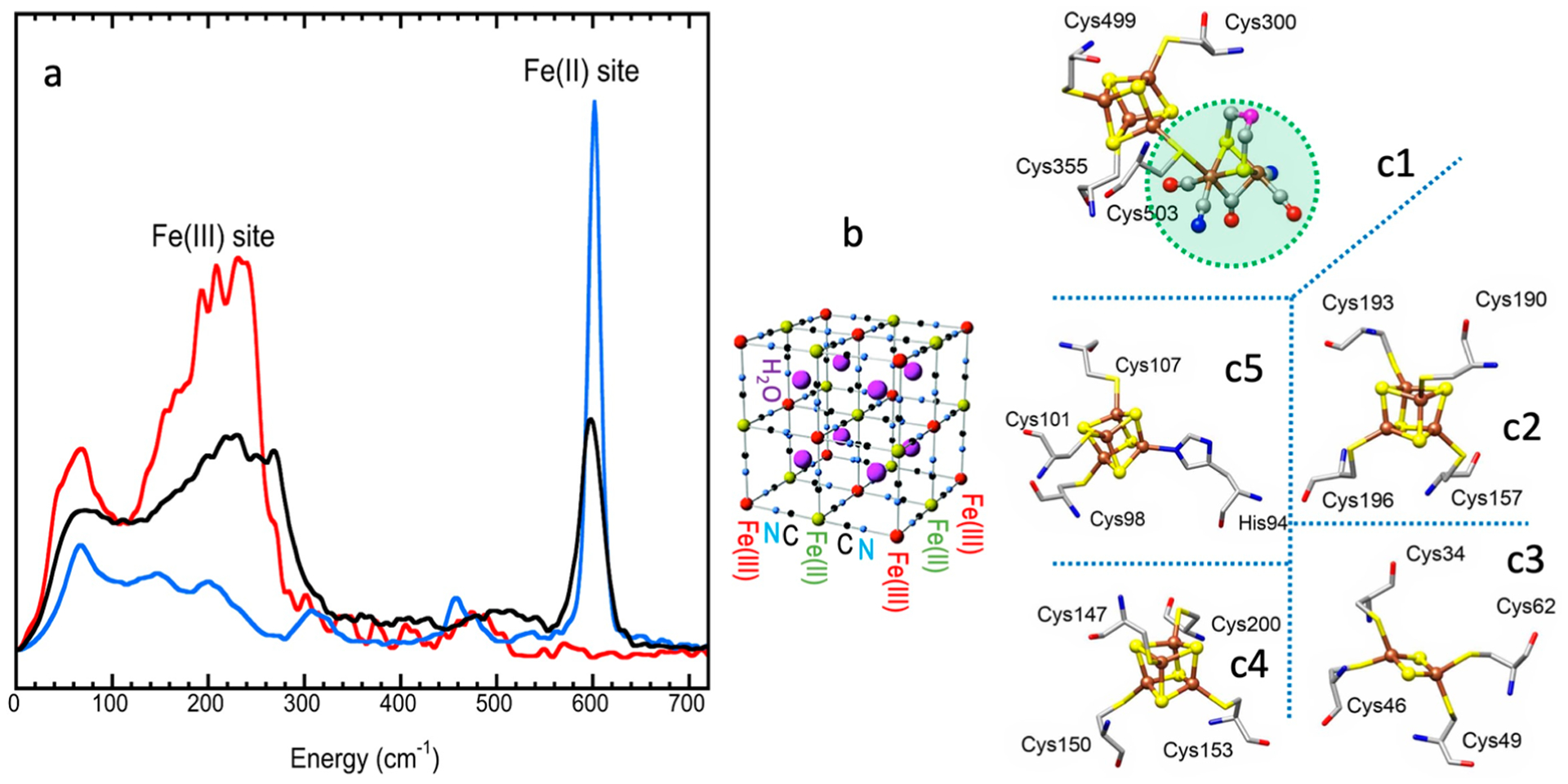
(**a**) NRVS for Prussian blue (PB) (black), K_2_[MgFe(II)(CN)_6_] (blue), and KFe(II)Co(III)(CN)_6_ (red); (**b**) structure of PB where red = Fe(III), yellowish green = Fe(II), black = C, blue = N, and purple = H_2_O; (**c**) iron clusters inside an FeFe hydrogenase in Figure 2a: the green shaded region is the [2Fe]H sub-cluster, while (**c1**) is the H-cluster; the rest are the proximal (**c2**), median (**c4**), and distal (**c5**) [4Fe4S] clusters and the [2Fe2S] cluster (**c3**). The brown atoms are iron, while the yellow ones are sulfur.

The NRVS spectra (a, black) and the crystal structures (b) for the Fe(II) and Fe(III) sites inside the Prussian blue (PB) complex Fe(III)_4_[Fe(II)(CN)]_3_·xH_2_O (x = 14−16) are illustrated in Figure 16. Due to its distinct Fe(II, S = 0) and Fe(III, S = 5/2) sites, the complex becomes one of the classical examples for evaluating various new high-resolution spectroscopies. For example, almost 20 years ago, Glatzel et al. published an article [103] demonstrating the site-selective EXAFS using high-resolution fluorescence detection of the K_β_ emission. The K_β_ fluorescence lines arising from the Fe(II, S = 0) and from the Fe(III, S = 5/2) sites were a little bit different in energy, while the high-resolution spectrometer could resolve their K_β_ fluorescence [103]. For NRVS, the strong interactions between Fe(II) and (CN) lead to a sharp Fe(II, S = 0)–CN peak at 602 cm^−1^ [16]. On the other hand, Fe(III) is more likely to have a loose interaction with several whole Fe(II)(CN)_6_ clusters around it and, therefore, has a broad hump between 150 and 300 cm^−1^ instead.

To illustrate the nature of these peaks, either the Fe(II, S = 0) (yellowish green in Figure 16b) or the Fe(III, S = 5/2) (red) site can be substituted with other metals via special synthetic methods, leaving only one site labeled with ^57^Fe. This approach creates two site-specific complexes: one is the KFe(II)Co(III)(CN)_6_ molecule with a [Co(CN)_6_] core structure and a loosely bound ^57^Fe(II) … [Co(CN)_6_] interaction, thus having a big bump at 150–300 cm^−1^ in the ^57^Fe NRVS spectrum (the red curve in Figure 16a), and the other is the (NH_4_)_2_[MgFe(II)(CN)_6_] [16] with a tight [^57^Fe(II)(CN)_6_] core structure which is loosely bound to Mg. The second complex, thus, has a strong NRVS peak at 602 cm^−1^ (blue) [16] but has almost no front bump in its ^57^Fe NRVS. These NRVS spectra adequately illustrated the different features from the original Fe(II) vs. Fe(III) sites inside PB, while also demonstrating the concept of site-selective labeling on particular iron site. As illustrated in Figure 16a, natural PB (black) has a medium peak at 602 cm^−1^ and a medium bump at 150–300 cm^−1^.

In the real science world, large molecules can have many different iron clusters. For example, the FeFe hydrogenase in Figure 2a can have five iron clusters, as illustrated in detail in Figure 16c1–c5. The different iron clusters can be grown separately via different cluster-making proteins, and the whole FeFe hydrogenase can be assembled at the end of the process. Therefore, people can select which iron cluster(s) to label in the corresponding production process, i.e., the [2Fe]_H_, the H-cluster, or any other FeS clusters in Figure 16. Site-selective labeling on one cluster enables the interesting features (e.g., the weak X–Fe–H bending peaks) to stand out from the unwanted background [30,44,45,47]. On the other hand, people have not realized site-selective labeling for NiFe hydrogenases so far because their clusters are not produced via different routes and, thus, cannot be manipulated.

## 6. Future Perspectives

### 6.1. Non-^57^Fe NRVS

**Figure 17.**
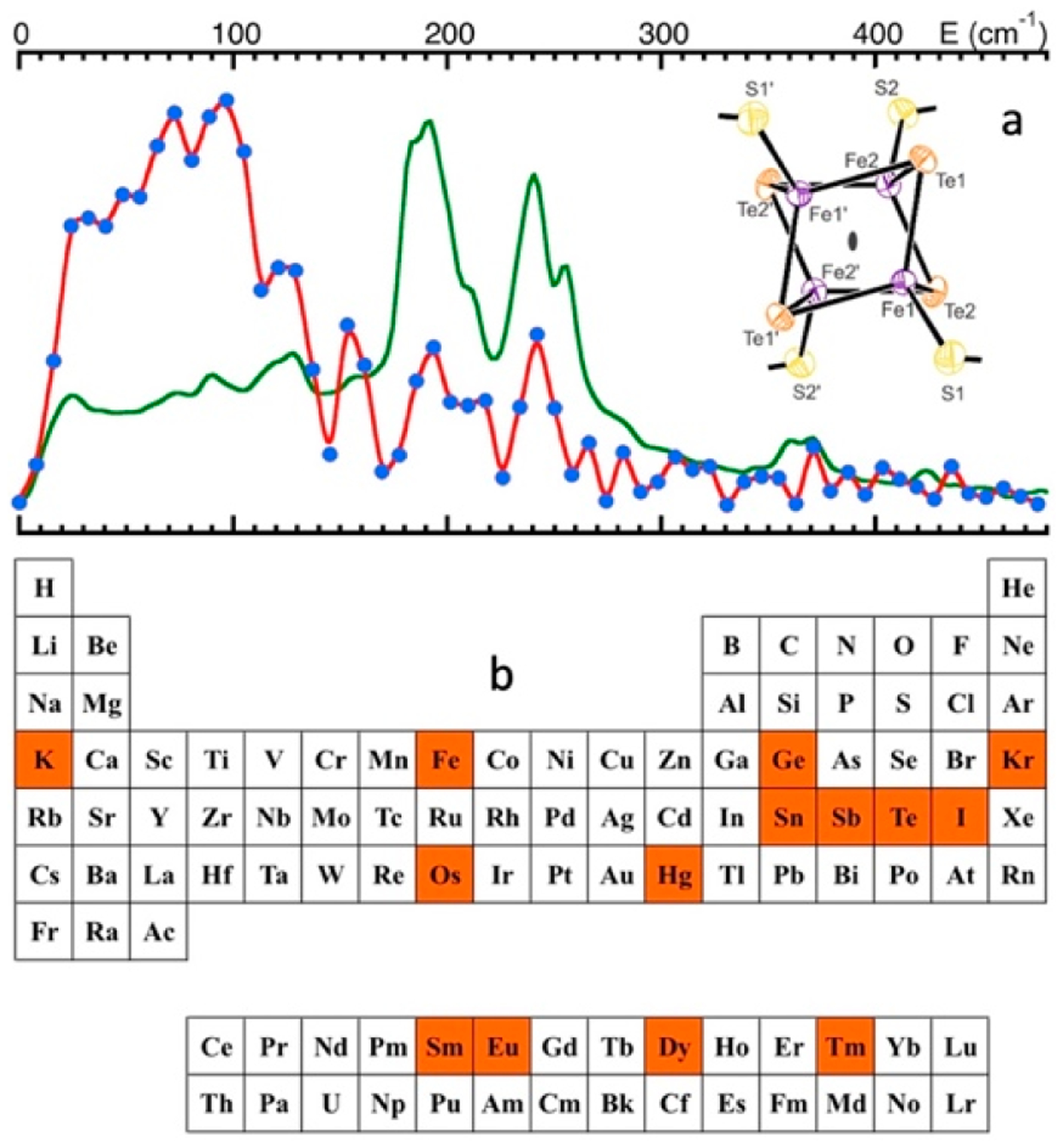
(**a**) ^57^Fe (green line) and ^125^Te (red line with blue dots) NRVS spectra for the same [4Fe4Te]^+^ cluster; (**b**) periodic table with the frequently studied NRS isotopes highlighted in orange.

Although the most extensively studied NRVS is with the ^57^Fe isotope, NRVS or NRS studies for non-^57^Fe isotopes exist—some studies are even before ^57^Fe NRVS [40]. In Figure 17a, we summarize the ^57^Fe and ^125^Te spectra for the same [Et_4_N]_3_[Fe_4_Te_4_(SPh)_4_] complex (or [4Fe4Te]^+^ for short) from a recent publication [39]. The central structure is shown in the insert.

Figure 17a outlines the overall conclusion that the ^57^Fe (green line) and the ^125^Te (red line with blue dots) NRVS spectra have the same corresponding peaks for this [4Fe4Te]^+^ cluster. In the original publication [39], both NRVS spectra and their structures were analyzed in detail and compared with regular [4Fe4S]^+^ NRVS. DFT calculations were also used to discuss the corresponding structural, electronic, and vibrational properties of this [4Fe4Te]^+^ ion. ^125^Te gives rise to a much noisier NRVS spectrum in comparison with ^57^Fe. In addition to possible differences in the experimental details, this difference is due to several principal factors: (1) a much lower incident flux at ^125^Te’s much higher nuclear resonant energy (35.5 keV for ^125^Te vs. 14.4 keV for ^57^Fe); (2) a much less effective time integration region due to its much shorter lifetime (3.5 ns vs. 98 ns, half lifetime). Nevertheless, this work provided a site-selective option for investigating specific S site(s) inside various biological sites. While Te is not an essential trace element in biology, certain Te compounds can be bio-metabolized and, thus, manipulated into various biological systems [104]. In addition, Te is a component for many advanced materials, such as thermoelectric materials, phase-change materials for data storage, superconductors, solar cells, and IR detectors [39]. Thus, ^125^Te NRVS provides a useful method to evaluate the structure and dynamics of these advanced materials.

There are many more NRVS-compatible isotopes across the periodic table, as shown in highlighted blockers in Figure 17b. Table 1 also summarizes a list of current NRS beamlines and their studied isotopes around the world.

Existence of a Mössbauer isotope is the necessary condition for an NRVS experiment but not a sufficient condition. In order to be suitable for an NRVS experiment, the isotope’s excitation energy should not be too high (so that the incident beam flux is still strong), the excited state lifetime should not be too short (at least several ns), and a proper HRM should exist. For different nuclear transitions, people have to use different HRMs (with different key crystal pairs). So far, there are many HRMs around the world which can provide high-resolution X-rays at energies of 5–30 keV. Some of them (not all of them) have an energy resolution at <3 meV, suitable for NRVS experiments. For example, at SPring-8 BL09XU, the HRMs for ^181^Ta and ^61^Ni have energy bandwidths at 10.5 meV and 60 meV, respectively, making them not suitable for NRVS studies (but suitable for synchrotron Mössbauer spectroscopy, compared to the aforementioned ^61^Ni for example [75]). Meanwhile, all other HRMs are suitable for NRVS. Most of these HRMs and associated NRVS facilities are moving to BL35XU this year.

There are many cases where NRVS on inorganic materials would be worthwhile to pursue, particularly for those systems where transport properties are affected by the vibrational structure of the material. This could include many solid-state electrochemical energy converters with ceramic electrolytes (oxygen/proton conductors, and lithium/sodium conductors). In a recent study, indication was found that particular vibrational modes propel, for example, the protons in yttrium-substituted barium zirconate [105]. In particular, the inelastic and quasi-elastic neutron scattering methods were instrumental in learning more about the charge carrier dynamics in the aforementioned systems, notwithstanding that the nature of the proton as a mobile species, activated by energy from the thermal bath [106], may still remain mysterious [107]. Element-specific vibrational spectra from NRVS might further shine light on the cooperative origin of proton transport, for example, with respect to questions raised about previous studies [108,109].

### 6.2. NRVS with Spatial Resolution

**Figure 18.**
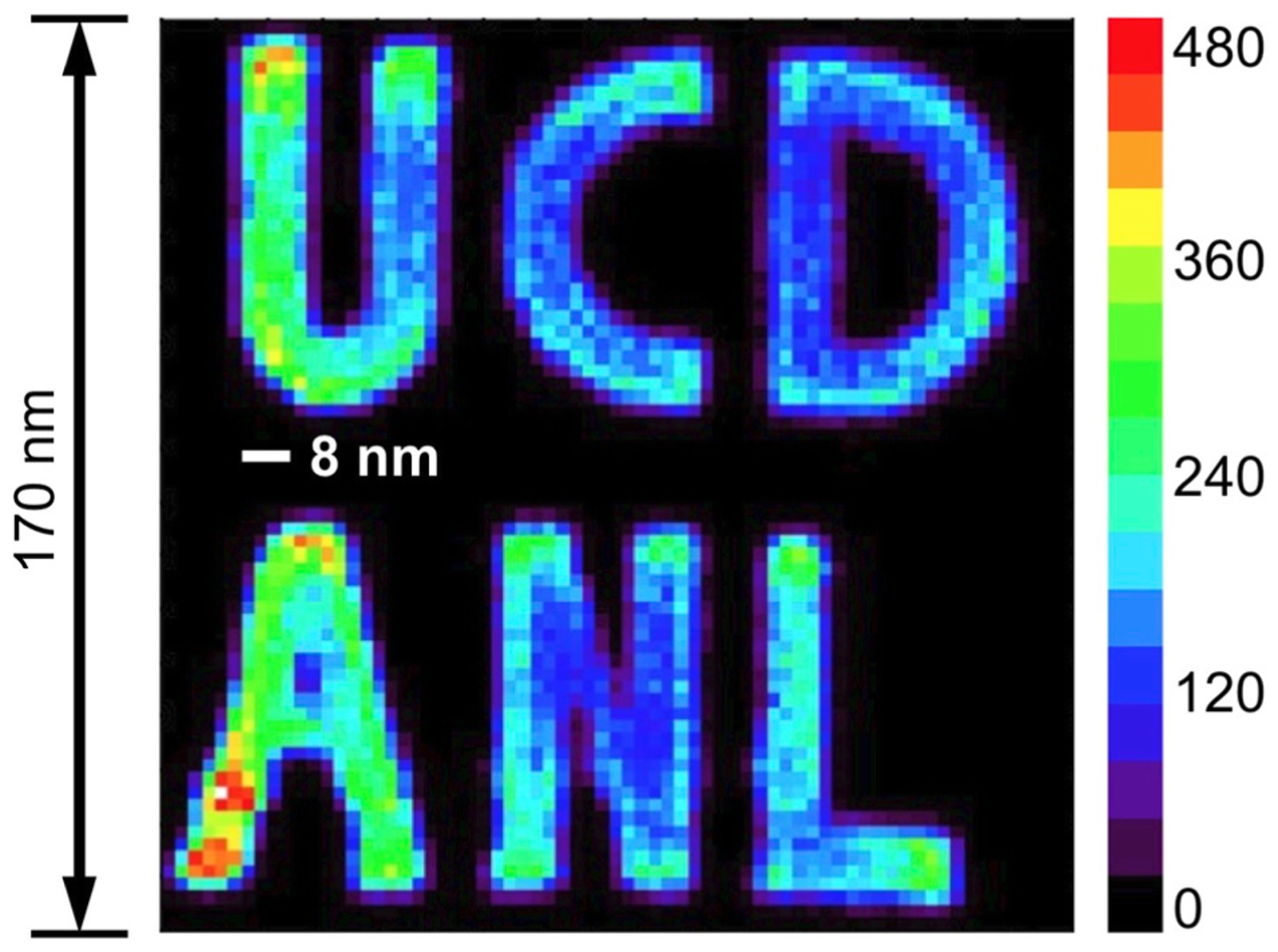
^57^Fe SMS image of a phantom recorded in transmission geometry at APS 03ID, cited from [111].

In the development of analytical tools in chemistry and materials science, including biological systems, there has always been interest in spatial resolution to the extent of imaging and microscopy. A scanning or tomography mode of NRVS is basically in sight. A Mössbauer scanning microprobe using a ^57^Co source was presented in 2008 and applied to iron-containing samples, with a spatial resolution of around 20 μm [110]. In 2012, a synchrotron-based Mössbauer spectromicroscopy (SMS) project was presented, applied to artefact phantom samples enriched with ^57^Fe and a meteorite sample with natural abundance of ^57^Fe [111], with a demonstrated spatial resolution of less than 10 nm, as shown in Figure 18. They also applied nuclear resonant incoherent X-ray imaging (NRIX imaging). This method collects incoherent nuclear resonant scattered intensity in the 4π direction and could provide information on the lattice dynamics. This may be important for understanding the electric transport properties of a specimen. From a more practical point of view, thick samples like rocks can be studied. Geological and geochemical samples, as well as rare specimens of astronomical origin, have been frequently studied with Mössbauer methods. The spatial resolution of these methods is still of great interest [112].

A not often appreciated property of analytical methods is their depth sensitivity. While we have just mentioned aspects of lateral spatial resolution, the resolution with respect to depth in a sample can also be interesting in geochemistry and in materials technology. Soft X-ray tools and X-ray photoelectron spectroscopy are synchrotron methods specifically used for surface-sensitive studies. Those are very important for elucidating problems relating to catalysis and electrochemical energy storage and conversion. A practical example for depth sensitivity in Mössbauer was shown in a paper by Fleischer et al. [113], with a genuine rock sample from Mars and a synthetic sliced sample. The latter should aid in mathematical simulation of the spectroscopic information obtained from the samples emitting 6.4 keV (hν_2_ in Figure 1a) and 14.4 keV (hν_1_) radiation (with different ratio at different depth) while excited with 14.4 keV. This spatial Mössbauer study paves an effective way to evaluate depth-dependent properties of natural materials, and synchrotron-based NRS and NRVS should have some roles in the future. The energy-dependent penetration (escape) depth of the photons is used here for obtaining the depth-specific information. This approach reminds us of an Mn 2p soft X-ray absorption and Mn Kβ emission spectroscopy study we did on battery electrode materials in order to learn about the oxidation state of the Mn at the surface and in the bulk [114].

Synchrotron X-rays can be focused to a few nanometers by zone plates [115], which have been used in scanning transmission X-ray micro spectroscopy (STXM) machines since the 1990s [116]. It would be interesting to further develop such methods for chemical contrast (or contrast in molecular structure and electronic structure) with Angstrom spatial resolution. Then, it could be possible to study the influence of particular ions in layered systems, such as battery [117] and fuel cell electrode materials or high-temperature superconductors, provided the layers are oriented enough to access them reasonably with the X-ray beam. The latter could apply, for example, to the iron bearing superconductors [89], the transition temperature of which depends on the spatial separation of these layers. As these superconductors contain iron, they have already been studied with Mössbauer spectroscopy. ^57^Fe or other isotope NRVS could provide a pinpoint tool to aid in the evaluation of their structural details.

### 6.3. NRVS at Future SR Sources

Building diffraction limit SR rings (DLSR) or upgrading the existing rings to DLSR has interested many scientists including NRVS users. In a DLSR, there are at least two major improvements in the incident X-ray beam: a much smaller horizontal beam divergence and a much smaller beam size. These properties further lead to (1) a higher beam brightness, (2) a higher throughput in HRM and/or other X-ray optics, (3) a higher spatial resolution in SMS due to the collimated beam, (4) a better beam–sample interaction (see Section 2.4, Figure 5b1 vs. b2 for details), (5) feasible-to-use small crystal samples (the high concentration and the orientation enhancement further strengthen the NRVS signal), and (6) feasible-to-measure NRVS in small and extreme environments including ultrahigh pressure, ultrahigh temperature, ultralow temperature, and ultrastrong magnetic field. As SR rings, as well as insertion devices, HRMs, and other X-ray optics, continue to improve, it is estimated that the NRVS or the NRS signal levels could have a 1–2 order of magnitude improvement with a DLSR ring. This will make it possible to use NRVS to examine some extremely weak features, which are still impossible to measure today. The examples include the Fe–D/H stretching vibrations inside a biological molecule or some extremely dilute intermediate biomolecules, such as Q intermediates in MMO enzymes.

Due to the possible huge flux surge, building an HRM with a 0.1–0.2 meV energy resolution (instead of 1 meV) is expected to occur. To avoid detector saturation, array detectors with 16, 32, or even more than 100 elements are being evaluated and/or planned. Radiation damage may also become a real concern as the beam flux reaches 10^11^–10^12^ photons/s.

The SR’s average emittance in the horizontal direction ε_0_ is inversely proportional to the third power of the number of turning magnets (N_d_) on the ring: ε_0_ ~ 1/N_d_^3^. Therefore, the basic idea for DLSRs is to install more turning magnets and reduce the turning angle of the moving electrons at each magnet. However, this will also greatly reduce the electron bunch interval, while measurement of NRVS or other nuclear scattering spectroscopy methods rely on the long electron bunch interval. Therefore, it will be difficult to perform NRVS or NRS measurements on an ideal DLSR ring, especially for the experiments targeting isotopes with long decay periods, e.g., ^57^Fe. The development direction of DLSR has, thus, drawn special attention from NRVS or NRS experimentalists, who hope the future DLSR can balance the DLSR properties and the long electron bunch interval. For example, Petra-III has an emittance of 1 nm·rad, reaching the door of a DLSR ring, and it still maintains operational mode with an ~150 ns time interval. Another proposal will be a 75 ns time interval (such as that at SKIF), which can keep inelastic scattering (such as NRVS) running as is.

If an ideal DLSR is to be built with densely packed electron bunches, all nuclear scattering experiments with long decay lifetime (e.g., ^57^Fe) will become difficult. In that case, the nuclear lighthouse effect provides an alternative detection of the time spectra for various nuclear scattering process, which can bypass the requirement for a long time interval.

**Figure 19.**
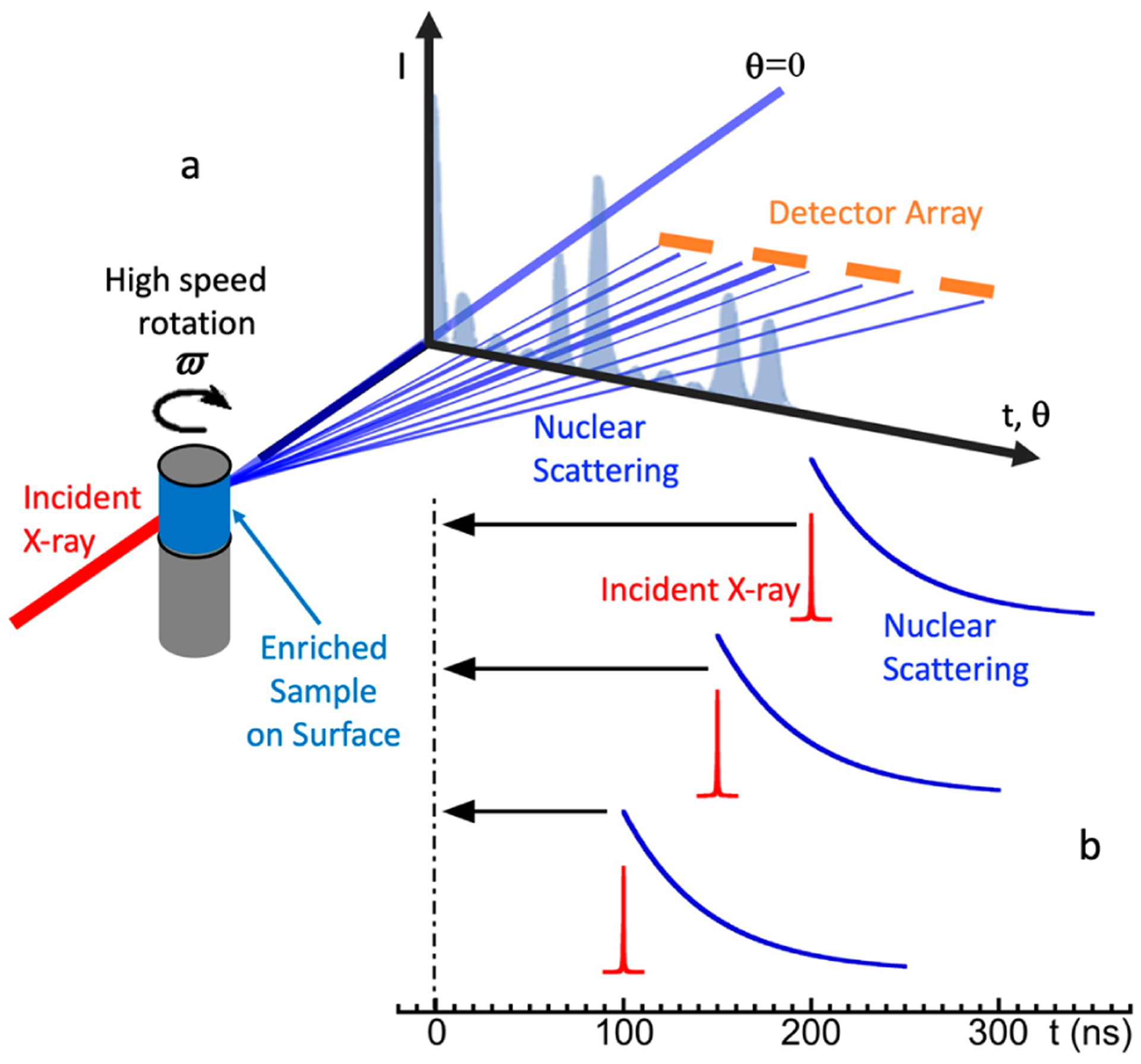
(**a**) An illustrative drawing for the concept of nuclear scattering detection using the lighthouse effect; (**b**) in the detection system using the lighthouse effect, the zero points for three X-ray pulses (red) at any time, along with their induced delayed nuclear scattering events (blue), can be recalibrated to the real t = 0 (the time when each X-ray pulse arrives) and then properly added together.

Figure 19a is conceptually duplicated from a previous publication by Roth et al. [118]. In such a typical apparatus, a high-resolution X-ray beam is irradiated to the smooth surface of a sample rotating at a super high angular velocity *ω*, and the nuclear scattering beam from the sample is deflected with the high-velocity rotation: (1) at the arrival of the incident X-ray photon, the scattering light is always in the direction of *θ* = 0 no matter what time the incident photon arrives; (2) after a specific time delay (t = t’) following the arrival of the incident photon, the nuclear scattering beam is deflected to a specific direction at *θ* = *ωt*’, where ω is the angular velocity of the sample. Intuitively speaking, this is a bit like an umbrella spinning off rain drops although their theoretical backgrounds are very different. With this apparatus, the task of collecting the scattered nuclear signals at different time delay (t’) will become the task of collecting the scattered signals at different *θ* = *ωt*’. In other words, the nuclear scattering time spectrum is mapped onto an angular distribution, which allows people to record with a position sensitive detector such as an APD array or a CCD without using timing electronics. This is equivalent to moving all three incident X-ray pulses (red spike) in Figure 19b to the zero position at t = 0 and moving the corresponding nuclear scattering signals (blue) accordingly. This is a very novel concept, and its characteristics allow people to continue nuclear scattering experiments in a real DLSR ring.

Of course, such an experiment requires an extremely flat/smooth sample, along with an extremely high angular velocity rotation. Although it sounds impossible for a biological or chemical sample, it seems possible for an alloy or fine materials science sample.

### 6.4. More Prospective Applications

It appears that the majority of NRVS studies so far have been carried out on chemical or biological samples, typically at temperatures where their biological function is more or less frozen. It is of general interest to study proteins not only in their genuine functional setting in situ, but also while in operation as a component of the photosynthetic apparatus, operando so to speak. The same holds, for example, for the aforementioned energy converters and storage devices, where charge transport may be mediated as a cooperative process by lattice vibrations. Depending on the power performance of such devices and, thus, the time constants of their functionality, the speed at which NRVS scans can be performed must be adjusted. The very slow transport processes might be captured with ease, albeit at the cost of beamtime. On the other hand, for the fast processes, substantial new technology at the storage rings and/or beamlines must be developed before real-time measurements can be performed. One alternative to this may be that the chemical processes in energy storage and conversion can be technically slowed down.

It is worthwhile to mention that the studied enzymes can be considered as the biological predecessors of manmade catalysts relevant in industrial chemistry. The application, therefore, includes catalysts for the oxygen and hydrogen evolution reactions, as well as ammonia synthesis, and many more. In the development of X-ray methods, the solution of the catalysis problems in industrial chemistry was parallel to the development of X-ray absorption spectroscopy. This includes the development of in situ and the operando methods in particular. Perhaps NRVS will undergo similar exciting development in the future.

We conclude this review with an example in photonics. It is a material characteristic that the ^57^Fe isotope does resonantly absorb 14.4 keV γ-rays. It is possible to induce an optical transparency with respect to the γ-rays by collective oscillations in the acoustic branch. Induced optical transparency (IOT) is a well-known phenomenon of photonics which may be caused, for example, by ionic vibrations [119]. The physical origin is the interference by mode coupling, which can experimentally be achieved in various ways, including optical cavities [120]. This physical concept has been extended to γ-rays, first by using a conventional [121] Mössbauer source and recently by using a synchrotron Mössbauer source (SMS) [122].

**Figure 20.**
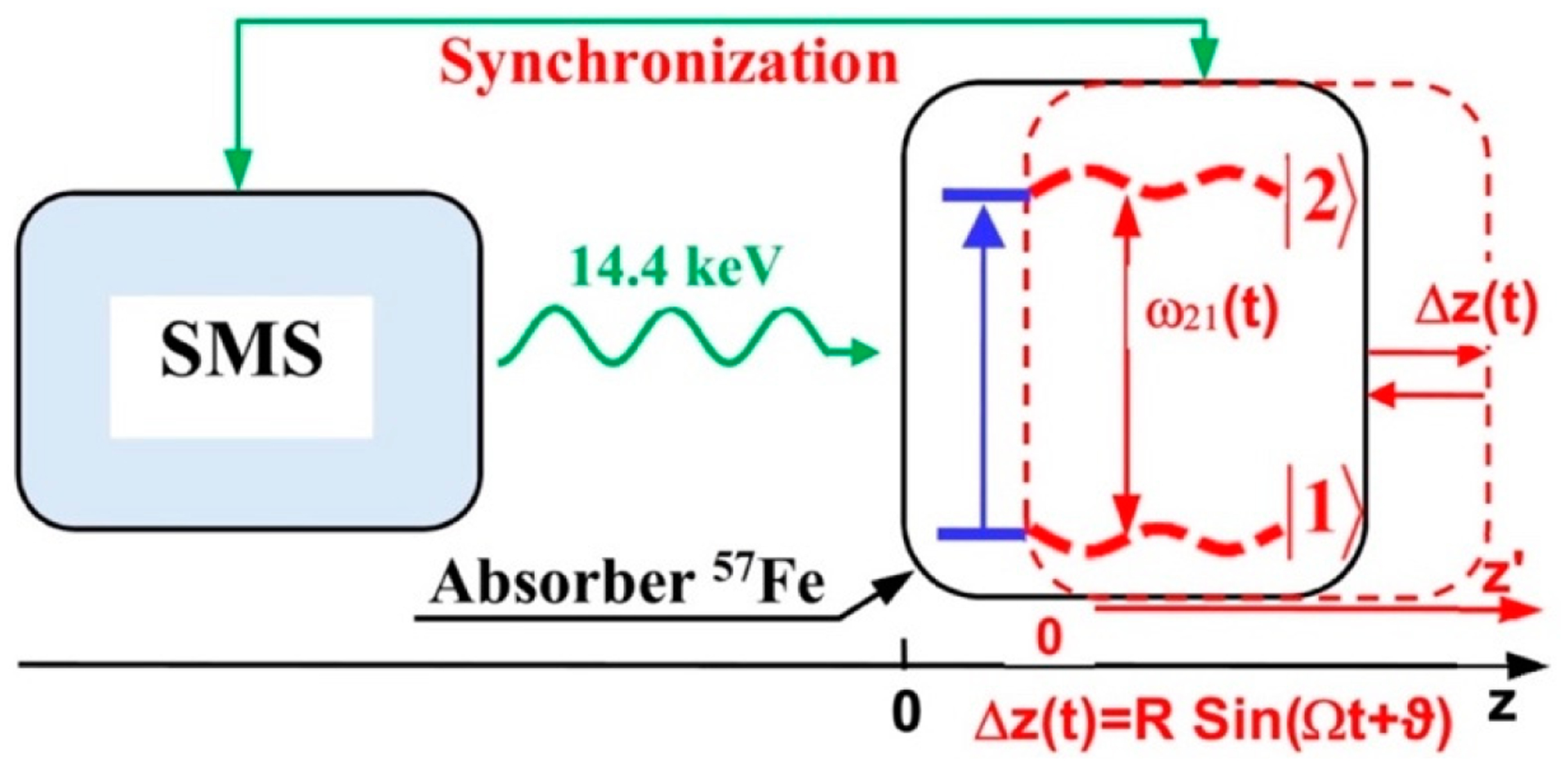
Energy scheme of the resonant interaction between 14.4 keV photons and vibrating absorber. Recoilless 14.4 keV photons (λ≈ 0.86 Å) emitted from the SMS (left side) resonantly interact with transition |1>→|2> of ^57^Fe nuclei during the propagation through the single-line ^57^Fe absorber (right side). They are resonantly absorbed in the motionless absorber (blue lines). Harmonic vibration of the absorber as a whole (piston-like vibration) with circular frequency Ω, amplitude R, and initial phase ϑ along the photon propagation direction (marked in red) or dual-tone vibration leads to periodic temporal variation in |1>→|2> transition frequency ω_21_ (t) (dashed red curves) due to the Doppler effect. The emission of photons can be locked to the initial phase of the absorber vibration, ϑ. The z-axis labels the laboratory reference frame, while the red z’-axis labels the reference frame of the vibrating absorber; Δz = z − z’. The figure and caption are cited from [122].

The experimental principle is illustrated in Figure 20 (reused from [122]). The ^57^Fe absorber, indicated by the black box on the right, receives the 14.4 keV photons from the synchrotron source SMS, while the absorber is moved away (actually, back and forth) from the SMS with a separation Δz(t) = R sin(ωt + θ) with an acoustic frequency ω. This causes a temporal variation of the transition states |1> to |2> with a modulation ω_21_(t) when the transmitted photons are synchronized with the absorber vibration. When the emission from SMS is locked to the initial phase of the acoustic absorber vibration, θ, a suppression of the absorption by a factor of 150 can be obtained [122]. This paves the way for the development of the acoustically controlled interface between hard X-ray photons and nuclear ensembles.

## Figures and Tables

**Table 1.** Some NRS beamlines and their studied isotopes.

SR/Beamlines	E (GeV)	Emittance (nm·rad)	Studied Isotopes
ESRF/18ID	6	4	^57^Fe, ^151^Eu, ^119^Sn, ^149^Sm, ^161^Dy, ^121^Sb, ^125^Te, ^129^Xe
APS/16ID	7	3.1	^57^Fe
APS/03ID	7	3.1	^57^Fe, ^151^Eu, ^119^Sn, ^83^Kr,^161^ Dy
SPring-8 /BL09XU	8	3.4	^181^Ta, ^57^Fe, ^151^Eu, ^149^Sm, ^119^Sn, ^40^K, ^125^Te,^121^Sb, ^61^Ni
SPring-8 /BL11XU	8	3.4	^57^Fe, ^121^Sb, ^149^Sm, ^40^K, ^158^Gd
SPring-8 /BL19LXU	8	3.4	^57^Fe
Petra-III/P01	6	1	^57^Fe, ^125^Te, ^119^Sn, ^121^Sb, ^193^Ir
